# COVID-19 epidemic in New York City: development of an age group-specific mathematical model to predict the outcome of various vaccination strategies

**DOI:** 10.1186/s12985-022-01771-9

**Published:** 2022-03-15

**Authors:** Miaolei Li, Jian Zu, Yue Zhang, Le Ma, Mingwang Shen, Zongfang Li, Fanpu Ji

**Affiliations:** 1 School of Mathematics and Statistics, Xi’an Jiaotong University, Xi’an, 710049 Shaanxi People’s Republic of China; 2 Department of Internal Medicine, The Second Affiliated Hospital of Xi’an, Jiaotong University, Xi’an, 710004 China; 3 Department of Infectious Diseases, The Second Affiliated Hospital of Xi’an Jiaotong University, 157 Xi Wu Road, Xi’an, 710004 Shaanxi Province People’s Republic of China; 4 School of Public Health, Health Science Center, Xi’an Jiaotong University, Xi’an, 710006 China; 5 National and Local Joint Engineering Research Center of Biodiagnosis and Biotherapy, The Second Affiliated Hospital of Xi’an Jiaotong University, Xi’an, 710004 China; 6 Key Laboratory of Environment and Genes Related To Diseases, Xi’an Jiaotong University, Ministry of Education of China, Xi’an, 710006 China; 7 Shaanxi Clinical Research Center of Infectious Diseases, Xi’an, 710006 China

**Keywords:** COVID-19, Age-structured model, Contact matrix, Vaccination strategies

## Abstract

**Background:**

Since December 14, 2020, New York City (NYC) has started the first batch of COVID-19 vaccines. However, the shortage of vaccines is currently an inevitable problem. Therefore, optimizing the age-specific COVID-19 vaccination is an important issue that needs to be addressed as a priority.

**Objective:**

Combined with the reported COVID-19 data in NYC, this study aimed to construct a mathematical model with five age groups to estimate the impact of age-specific vaccination on reducing the prevalence of COVID-19.

**Methods:**

We proposed an age-structured mathematical model and estimated the unknown parameters based on the method of Markov Chain Monte Carlo (MCMC). We also calibrated our model by using three different types of reported COVID-19 data in NYC. Moreover, we evaluated the reduced cumulative number of deaths and new infections with different vaccine allocation strategies.

**Results:**

Compared with the current vaccination strategy in NYC, if we gradually increased the vaccination coverage rate for only one age groups from March 1, 2021 such that the vaccination coverage rate would reach to 40% by June 1, 2021, then as of June 1, 2021, the cumulative deaths in the 75–100 age group would be reduced the most, about 72 fewer deaths per increased 100,000 vaccinated individuals, and the cumulative new infections in the 0–17 age group would be reduced the most, about 21,591 fewer new infections per increased 100,000 vaccinated individuals. If we gradually increased the vaccination coverage rate for two age groups from March 1, 2021 such that the vaccination coverage rate would reach to 40% by June 1, 2021, then as of June 1, 2021, the cumulative deaths in the 65–100 age group would be reduced the most, about 36 fewer deaths per increased 100,000 vaccinated individuals, and the cumulative new infections in the 0–44 age group would be reduced the most, about 17,515 fewer new infections per increased 100,000 vaccinated individuals. In addition, if we had an additional 100,000 doses of vaccine for 0–17 and 75–100 age groups as of June 1, 2021, then the allocation of 80% to the 0–17 age group and 20% to the 75–100 age group would reduce the maximum numbers of new infections and deaths simultaneously in NYC.

**Conclusions:**

The COVID-19 burden including deaths and new infections would decrease with increasing vaccination coverage rate. Priority vaccination to the elderly and adolescents would minimize both deaths and new infections.

**Supplementary Information:**

The online version contains supplementary material available at 10.1186/s12985-022-01771-9.

## Introduction

The COVID-19 has rapidly spread to more than 200 countries and regions around the world and has caused more than 166 million cases and 3.4 million deaths worldwide as of May 23, 2021 [1–3]. The COVID-19 pandemic posed a serious threat to the physical and mental health of people and had severe consequences for the economic development around the world [4–8]. With the rapid development of vaccines, many countries in the world had begun vaccination, meanwhile, new SARS-CoV-2 variants that are designated as Variants of Concern (such as B.1.1.7 (Alpha), B.1.351 (Beta), P.1 (Gamma), B.1.617.2 (Delta) and B.1.1.529 (Omicron) or variants of interest (VOI) (such as B.1.526 (Iota)) by WHO had appeared during the COVID-19 pandemic, these variants might help to evade current antibody therapy and vaccine protection, which brought some challenges to the current prevention and treatment of COVID-19 [9, 10]. As one of the epicenters of the United States, the COVID-19 pandemic in NYC was particularly serious. On December 14, 2020, NYC began applying the first batch of severe acute respiratory syndrome coronavirus 2 (SARS-CoV-2) vaccines [11]. However, the shortage of vaccines was and is currently an inevitable problem in NYC and other countries [12, 13]. Therefore, the allocation of COVID-19 vaccines to different age groups is an important issue which needs to be addressed as a priority [14, 15].

Currently, several mathematical models attempting to describe and estimate the transmission dynamics of COVID-19 with vaccination have been developed [16–24]. Table 1 summarizes different mathematical models used to describe the dynamics of the COVID-19 pandemic. Matrajt, et al. proposed an age-structured compartmental model with 16 age groups to explore the optimal vaccine distribution with various vaccine effectiveness and vaccine coverage, they showed that distributing vaccines to the elderly age groups would be more effective in reducing the deaths despite of low vaccine effectiveness in this population, and then switching to allocate vaccine to the young age groups with higher vaccine effectiveness would more conducive to epidemic control [19]. Iboi and Ngonghala et al. established a deterministic mathematical model of COVID-19, which showed that the threshold of herd immunity in the vaccine coverage rate to eliminate the COVID-19 pandemic in the US was 82% [20]. According to our previous study [21], the COVID-19 pandemic could be controlled in 4 states in the US when their vaccine coverage rate reached to 48–78%. However, few studies assessed the impact of age-specific vaccination on reducing the prevalence of COVID-19 combining with the reported COVID-19 data in NYC. Therefore it was considered necessary to develop further research including a mathematical model with age structure to evaluate the impact of age-specific vaccination.

**Table 1:** Summary of different mathematical models used to describe the dynamics of the COVID-19 pandemic

Authors	Model framework	Strengths	Limitations
Acuña-Zegarra et al. [16]	$SEI_{S} I_{A} RDV$	This study used optimal control methods with mixed constraints to evaluate different vaccination strategies to minimize the burden of COVID-19 pandemic, which could help policy decision makers design vaccination plans for the homogeneous population	This study did not consider the age structure or heterogeneity of the population
Libotte et al. [17]	$SIR$	This study developed a method to solve the inverse problem to determine the parameters of the SIR model, and considered the single- and multi-objective optimization environment to determine the best vaccination strategy for the COVID-19 pandemic	This study did not consider the age structure or heterogeneity of the population
Choi et al. [18]	$SVEPI_{S} I_{A} H^{M} H^{S} DR$	This study established an age-structured mathematical model and combined actual epidemiological data in Korea to evaluate vaccination strategies on the infection incidence and mortality for each age group under different levels of social distance	This study did not consider the priority vaccination for essential workers which had been planned by the Korean government
Matrajt et al. [19]	$SEPI_{S} I_{A} HH_{C}R$	This study used a mathematical model of age structure and combined optimization algorithms to evaluate different combinations of vaccine effectiveness and vaccination coverage for four different indicators (minimize deaths, minimize symptomatic infections, maximum non-ICU and minimize ICU hospitalizations)	This study assumed that asymptomatic and symptomatic infections had the same immunity. However, asymptomatic individuals may have a weaker immune response
Iboi et al. [20]	$S_{u} S_{v} E_{1} E_{2} I_{S} I_{A} HR$	This study evaluated the impact of vaccines on the control of COVID-19 in the United States based on a deterministic mathematical model, and derived the expression for the vaccine-induced herd immunity threshold	This study did not consider the age structure or heterogeneity of the population
Shen et al. [21]	$SVEI_{A} I_{1} I_{2} T_{1} T_{2} DR$	This study established a deterministic mathematical model, and evaluated the required vaccine effectiveness and vaccination coverage rate to suppress the COVID-19 pandemic, when the social contact returned to pre-pandemic normal levels and the face mask use was reduced	This study did not consider the age structure or heterogeneity of the population
Bubar et al. [22]	$SEIR$	This study used an age structured model to evaluate the impact of five COVID-19 vaccine priority strategies on cumulative incidence, mortality, and years of life lost	Due to the lack of direct measurement data, this study extrapolated the contact matrix to people over 80
Foy et al.[23]	$SVEI_{S} I_{A} QRD$	This study used an age structured model to investigate the impact of four age-based vaccination strategies on the infections and cumulative deaths, they concluded that allocating COVID-19 vaccines to older age groups (> 60 years) was the optimal in all scenarios considered regardless of vaccine efficacy, dispensation speed, force of infection	This study assumed that some model parameters that may vary with age and time, such as the latent period, the force of infection and the recovery rate to be constant due to the lack of clear data
Buckner et al. [24]	$SV_{P} V_{F} EPI_{S} I_{A} RD$	This study used an age structured model to solve for optimal strategies to allocate the limit COVID-19 vaccines to essential workers that minimizes the number of total deaths, years of life lost, or infections, they concluded that prioritizing the limit COVID-19 vaccines to older essential workers can better reduce mortality, and prioritizing the limit COVID-19 vaccines to younger essential workers can better control spread	This study did not consider the seasonality of contact rates for children in the scenarios where schools are modeled as closed. This may have limited impact on the optimal solutions

Here, the description of different compartments were as follows: susceptible individuals $S$; exposed individuals $E$; symptomatic infected individuals $I_{S}$; asymptomatic infected individuals $I_{A}$; recovered individuals $R$; dead individuals $D$; vaccinated individuals $V$; infected individuals $I$; pre-symptomatic infectious individuals $P$; hospitalized individuals with mild symptoms $H^{M}$; hospitalized individuals with severe symptoms $H^{S}$; hospitalized individuals $H$; hospitalized individuals who require intensive care $H^{C}$; unvaccinated susceptible individuals $S_{u}$; vaccinated susceptible individuals $S_{v}$; early-exposed individuals (i.e., newly-infected individuals who are not yet infectious) $E_{1}$; pre-symptomatic infectious individuals (i.e., exposed individuals who are close to surviving the incubation period and are shedding virus) $E_{2}$; undiagnosed infections with mild symptoms $I_{1}$; undiagnosed infections with severe symptoms $I_{2}$; diagnosed infections with mild symptoms $T_{1}$; diagnosed infections with severe symptoms $T_{2}$; self-isolated individuals $Q$; individuals vaccinated and protected $V_{P}$, individuals vaccinated but unprotected $V_{F}$.

In this study, we aimed to estimate the impact of age-specific vaccination on reducing the prevalence of COVID-19 and explore the best vaccination strategy. Particularly, we established an age-structured mathematical model with 5 age groups, and assessed the reduced cumulative number of deaths and new COVID-19 infections with different vaccine allocation strategies. These results can be used by public health physicians and policy decision makers in NYC and other countries and regions to formulate vaccination program.

## Methods

### Data sources

We collected three different types of reported COVID-19 data in 5 age groups (0–17, 18–44, 45–64, 65–74 and 75–100) in NYC from the official website of New York, specifically including cumulative confirmed cases (Additional file 3: Table S1, columns 2–6), cumulative deaths (Additional file 3: Table S1, columns 7–11) and cumulative hospitalizations (Additional file 3: Table S1, columns 12–16) from March 24, 2020 to February 28, 2021 [25, 26]. These reported COVID-19 data were used to calibrate our age-structured mathematical model and estimate the unknown parameters and initial values in the model. In addition, some new SARS-CoV-2 variants also had appeared during the COVID-19 pandemic in NYC. One of the variants of interest (VOI), B.1.526 (Iota) and one of the Variants of Concern (VOC), B.1.1.7 (Alpha), had appeared in NYC in later December 2020 [27–29]. The percent of NYC COVID-19 cases tested for variant viruses on January 2, February 27 2021 were 2% and 5% approximately. Currently, there is no clear evidence to draw definite conclusions on the characteristics of the new SARS-CoV-2 variants including the VOC or VOI, therefore, for simplicity, we had not considered the impact of these new SARS-CoV-2 variants.

### Model structure and assumptions

**Fig. 1:**
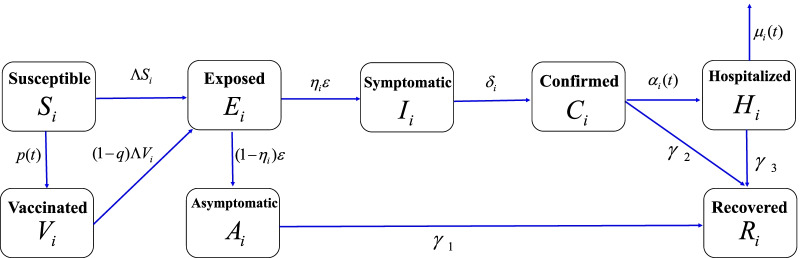
Flow diagram of the age-structured mathematical model for COVID-19 in NYC. The total population in NYC were divided into 5 age groups (0–17, 18–44, 45–64, 65–74 and 75–100 years). The sub-population in each age group $i$ in NYC were further divided into eight compartments: susceptible individuals $S_{i} (t)$; vaccinated individuals $V_{i} (t)$; exposed individuals $E_{i} (t)$; infected but asymptomatic individuals $A_{i} (t)$; infected and symptomatic individuals $I_{i} (t)$; confirmed individuals who stayed at home $C_{i} (t)$; hospitalized cases $H_{i} (t)$ and recovered cases $R_{i} (t)$. The details of the force of infection $\Lambda$ were provided in the Supplementary Text 1 (model formulation). The susceptible individuals $S_{i} (t)$ would become vaccinated individuals when they were vaccinated. The vaccination coverage rate was $p(t)$ and we assumed the vaccination coverage rate was a logistic function, i.e., $p(t) = \frac{{p_{\max } p_{0} }}{{p_{0} - (p_{0} - p_{\max } )\exp ( - rt)}}$, where $p_{\max }$ was the maximum vaccination coverage rate, $p_{0}$ was the initial vaccination coverage rate and $r$ was the growth rate of vaccination in NYC. The effectiveness of vaccine for COVID-19 was $q$. The incubation period of exposed individuals was ${1 \mathord{\left/ {\vphantom {1 \varepsilon }} \right. \kern-\nulldelimiterspace} \varepsilon }$. The recovery rate of asymptomatic infections in the free environment, confirmed cases and hospitalized cases were $\gamma_{1}$, $\gamma_{2}$, and $\gamma_{3}$, respectively. The proportion of symptomatic infections in age group $i$ was $\eta_{i}$, the transfer rate from symptomatic individuals to confirmed cases in age group $i$ was $\delta_{i}$, the transfer rate from confirmed cases to hospitalized cases in age group $i$ was $\alpha_{i} (t)$, and the death rate in age group $i$ was $\mu_{i} (t)$. Here, we assumed that $\alpha_{i} (t)$ and $\mu_{i} (t)$ were exponentially decreasing functions. More details were provided in the Additional file 1: Text 1 (model formulation)

According to the transmission mechanism of COVID-19 and the actual prevention and control strategy for the COVID-19 pandemic in NYC, we divided the total population in NYC into 5 age groups (0–17, 18–44, 45–164, 65–74 and 75–100 years) and established a susceptible-vaccinated-exposed-asymptomatic-symptomatic-confirmed-hospitalized-recovered (SVEI_A_I_S_CHR) model with an age structure at the population level (the detailed model formulation was described in the Additional file : Material for model formulation). We considered the exposed compartment because there was an incubation period from time of infection to the time of onset (first appearance) of symptoms when people were infected by SARS-CoV-2. Susceptible individuals and vaccinated individuals could get infected by contacts with the exposed individuals, and then the exposed individuals could progress to the infectious compartment with symptoms or asymptomatic compartment during the incubation period. We used the contact matrix to describe the contact differences among different age groups. Figure 1 showed the flow chart of our age-structured mathematical model.

### Model calibration

We first used the method of Markov chain Monte Carlo (MCMC) to estimate the unknown parameters of the age-structured mathematical model without vaccination (including the per-capita transmission rate, contact rates, the proportion of symptomatic infections, transfer rate from symptomatic individuals to confirmed cases, transfer rate from confirmed cases to hospitalized cases, death rate of hospitalized cases, recovery rate of asymptomatic infections, relative transmission strength of exposed, asymptomatic and confirmed individuals to the symptomatic individuals, increased proportion the contact rate from June 8, 2020 to September 20, 2020, and increased proportion the contact rate from September 21, 2020 to December 13, 2020) and some initial values (including the exposed, asymptomatic and symptomatic individuals), and calibrated the age-structured mathematical model without considering the vaccination by using the three different types of reported COVID-19 data in 5 age groups from March 24, 2020 to December 13, 2020 [30–32]. Additional file 3: Table S2 described the estimated initial values and parameters as well as their 95% confidence intervals. By comparing the estimated values in our model with the three different types of reported COVID-19 data in 5 age groups in NYC, we found that the estimated and reported values fitted very well (Additional file 2: Figs. S1–3).

Then, we further used the MCMC method to estimate the unknown parameters of the age-structured mathematical model with vaccination. We assumed that the vaccination coverage rate $p(t)$ was increased with a logistic growth, i.e., where $p_{\max }$ was the maximum vaccination coverage rate and was assumed to be 95%, $r$ was the vaccination rate which was unknown that needed to be estimated, $p_{0}$ was the initial vaccination coverage rate in NYC and was assumed to be equal to the vaccination coverage rate in New York State on December 14, 2020 based on the available vaccination data, December 14, 2020 was chosen as the initial time for vaccination (see the detailed parameter estimation in the Additional file 1: Text 1 (model formulation)). So we estimated the vaccination rate and some parameters that may be affected by vaccination (including the hospitalization rate, the death rate of hospitalized cases, the proportion of symptomatic infections and the transfer rate from symptomatic infections to confirmed cases) and calibrated the age-structured mathematical model with vaccination by using the three different types of reported COVID-19 data in 5 age groups from December 13, 2020 to February 28, 2021, the estimated and reported values fitted very well (Additional file 2: Figs. S1–3). Additional file 3: Table S3 described the estimated parameters as well as their 95% confidence intervals. Thus, our model and the estimated parameters and initial values were credible and could be used to further assess the impact of age-specific vaccination on reducing the prevalence of COVID-19.

### Impact of age-specific vaccination strategies

In order to explore the impact of age-specific vaccination for the COVID-19 pandemic in NYC, we simulated the following three scenarios.

1. First, starting from March 1, 2021, we increased the vaccination rate for only one age group (0–17 age group, 18–44 age group, 45–64 age group, 65–74 age group, or 75–100 age group) such that as of June 1, 2021, the vaccination coverage rate in this age group would reach 40%, 60%, and 80% [p(169) = 40, 60, 80%], respectively, and we evaluated the reduced cumulative number of deaths and new infections in this age group. Besides, we calculated the increased number of vaccinated individuals in this age group as of June 1 after increasing the vaccination coverage rate from March 1. Since the number of vaccinated individuals and the reduced cumulative number of deaths and new infections for each age group was different, in order to explore which age group should be given priority for COVID-19 vaccine when vaccines were limited, we performed normalization processing. We compared which age group would reduce the cumulative number of deaths and new infections most under the same number of immunized individuals. In this way, we calculated the reduced cumulative number of deaths and new infections per 100,000 vaccinated individuals in each age group. Based on the normalization results, finally, we selected the age group which reduced the cumulative number of deaths most as age group A and the age group which reduced the cumulative number of new infections most as age group B.
2. Second, starting from March 1, 2021, we increased the vaccination rate for two age groups (here we considered 0–17 age group and 18–44 age group, 18–44 age group and 45–64 age group, and 65–74 age group and 75–100 age group, that is, 0–44 age group, 18–64 age group, and 65–100 age group) such that as of June 1, 2021, the vaccination coverage rate in these two age groups would reach to 40%, 60%, and 80%, respectively, and we evaluated the reduced cumulative number of deaths and new infections in these two age groups. Besides, we calculated the increased number of vaccinated individuals in these two age groups as of June 1 after increasing the vaccination coverage rate from March 1.Through normalization, we compared which two age groups reduce the cumulative deaths and new infections most under the same number of immunized individuals. In this way, we calculated the reduced cumulative number of deaths and new infections per 100,000 vaccinated individuals in these two age groups.
3. Third, in order to ensure the greatest reduction in the number of cumulative deaths and new infections simultaneously, based on the results of the first scenario, we considered the age group which reduced the cumulative number of deaths most as age group A and the age group which reduced the cumulative number of new infections most as age group B. Then starting from March 1, 2021, based on the current vaccination rate of 5 age groups, we allocated a batch of additional vaccines to the age group A and age group B such that as of June 1, 2021, an additional 100,000 people in these two age groups would be vaccinated. We reallocated these additional vaccines to age group A and age group B in proportion, and evaluated four vaccine allocation schemes (allocation scheme 1: age group A accounted for 20% and age group B accounted for 80%; allocation scheme 2: age group A accounted for 40% and age group B accounted for 60%; allocation scheme 3: age group A accounted for 60% and age group B accounted for 40%; and allocation scheme 4: age group A accounted for 80% and age group B accounted for 20%). We calculated the reduced cumulative number of deaths and new infections in these four vaccine allocation schemes. To compare which vaccine allocation scheme would reduce the cumulative number of deaths and new infections most simultaneously in NYC, we performed normalization processing. Specifically, we mapped the reduced cumulative number of deaths in the four vaccine allocation schemes into the range of 0 to 1. The largest reduced cumulative number of deaths was regarded as 1, and the reduced cumulative number of deaths in the remaining three vaccine allocation schemes was scaled proportionally. Similarly, the largest reduced cumulative number of new infections was also regarded as 1, and the reduced cumulative number of new infections in the remaining three vaccine allocation schemes was scaled proportionally. Adding the normalized results for the reduced cumulative number of deaths and new infections in each vaccine allocation scheme, then we obtained the optimal vaccine allocation strategy.

## Results

### Impact of vaccination for one age group

#### Reduction of cumulative deaths

**Fig. 2:**
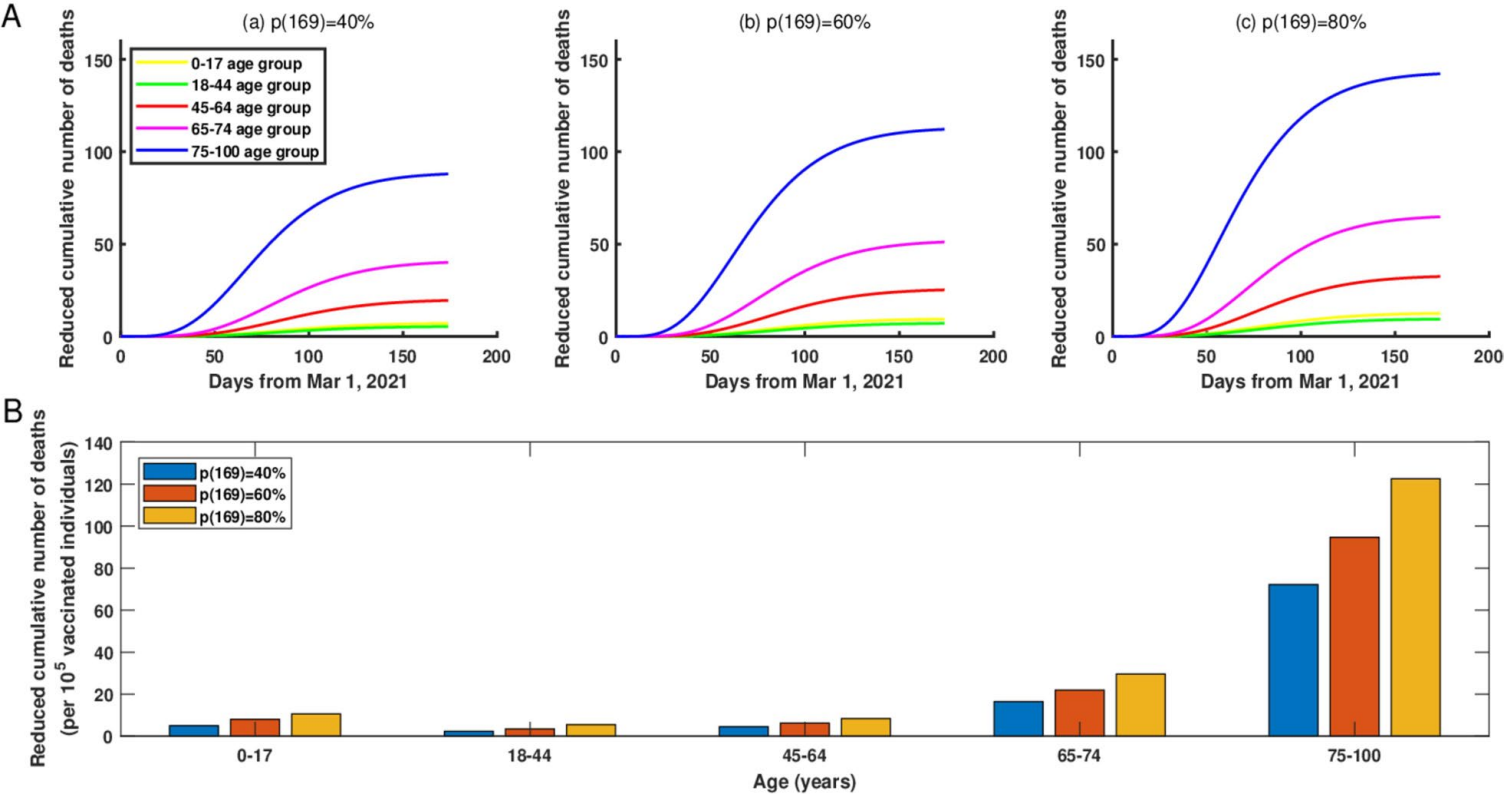
**A**The reduced cumulative number of deaths in NYC if starting from March 1, 2021, the vaccination coverage rate for only one age group was gradually increased and would reach to 40%, 60%, and 80% by June 1, 2021, respectively. (a) The vaccination coverage rate was 40%. (b) The vaccination coverage rate was 60%. (c) The vaccination coverage rate was 80%. **B** The reduced cumulative number of deaths per increased 100,000 vaccinated individuals in only one age group if the vaccination coverage rate was increased to 40%, 60%, and 80% by June 1, 2021, respectively

Compared with the current vaccination strategy, if starting from March 1, 2021, the vaccination coverage rate for only one age group (0–17age group, 18–44 age group, 45–64 age group, 65–74 age group, or 75–100 age group) was gradually increased to 40% by June 1, 2021, then the top three age groups with the greatest reduction in cumulative deaths were 75–100, 65–74 and 45–64 age groups as of June 1, 2021, and the reduced cumulative deaths were 63, 23 and 10, respectively (Figure 2A (a)). However, if the vaccination coverage rate for only one age group was gradually increased to 80% by June 1, 2021, then the top three age groups with the greatest reduction in cumulative deaths were still 75–100, 65–74 and 45–64 age groups as of June 1, 2021, and the corresponding reduced cumulative deaths were 111, 42 and 19, respectively (Figure 2A (c)).

In addition, the increased vaccination coverage rate would lead to an obvious reduction in the cumulative number of deaths in NYC (Fig. 2B (a–c)). Specifically, if starting from March 1, 2021, the vaccination coverage rate in the 75–100 age group was increased up to 40%, 60%, and 80% by June 1, 2021, then through normalization, we obtained the reduced cumulative number of deaths in NYC were 72, 95, and 123 per increased 100,000 vaccinated individuals, respectively. Moreover, from Fig. 2B, we can see that increasing the vaccination coverage rate for middle and elderly age groups would reduce the cumulative number of deaths even more in NYC.

#### Reduction of cumulative new infections

**Fig. 3:**
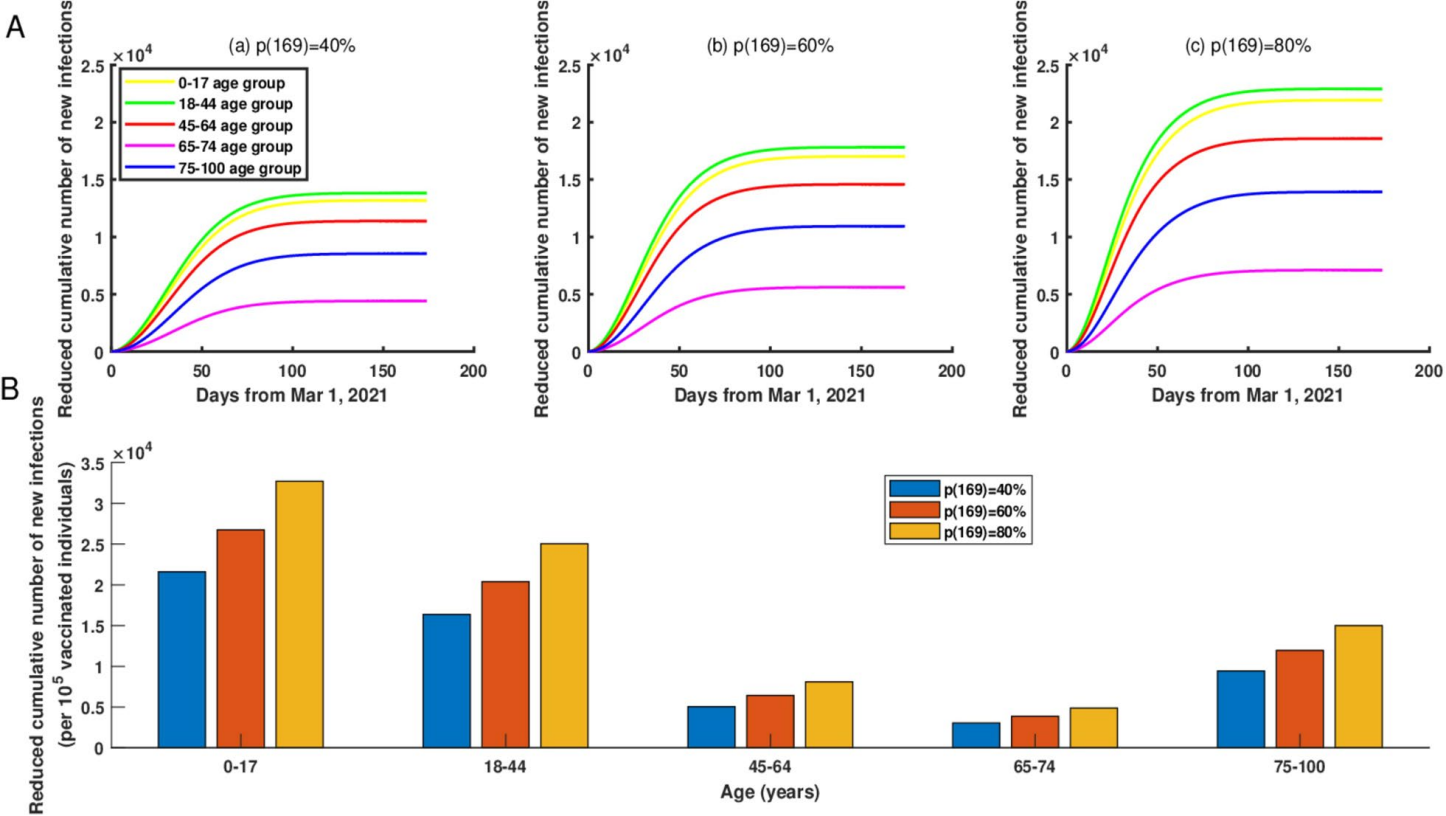
**A** The reduced cumulative number of new infections in NYC if starting from March 1, 2021, the vaccination coverage rate for only one age group was gradually increased and would reach to 40%, 60%, and 80% by June 1, 2021, respectively. (a) The vaccination coverage rate was 40%. (b) The vaccination coverage rate was 60%. (c) The vaccination coverage rate was 80%. **B** The reduced cumulative number of new infections per increased 100,000 vaccinated individuals in only one age group if the vaccination coverage rate was increased to 40%, 60%, and 80% by June 1, 2021, respectively

If we gradually increased the vaccination coverage rate for only one age group (0–17 age group, 18–44 age group, 45–64 age group, 65–74 age group, or 75–100 age group) to 40% by June 1, 2021, then compared with the current vaccination strategy, the top three age groups with the greatest reduction in cumulative new infections were 18–44, 0–17 and 45–64 age groups, and the reduced cumulative new infections were 13463, 12807 and 11073, respectively (Figure 3A (a)). Furthermore, if we gradually increased the vaccination coverage rate for only one age group to 60% by June 1, 2021, then, as of June 1, 2021, the top three age groups with the greatest reduction in cumulative new infections were still 18–44, 0–17 and 45–64 age groups, and the reduced cumulative new infections were 17452, 16628 and 14265, respectively (Figure 3A (b)). In addition, from Figure 3A (c), we can see that the increased vaccination coverage rate would lead to an obvious reduction in the cumulative number of new infections in each group.

In addition, if the vaccination coverage rate in the 0–17 age group was increased to 40%, 60%, and 80% by June 1, 2021, then through normalization, we obtained the reduced cumulative number of new infections were 21,591, 26,746, and 32,708 per increased 100,000 vaccinated individuals, respectively. Furthermore, increasing the vaccination coverage rate for 0–17 and 18–44 age groups would reduce the cumulative number of new infections even more in NYC (Fig. 3B (a–c)).

### Impact of vaccination for two age groups

#### Reduction of cumulative deaths

**Fig. 4:**
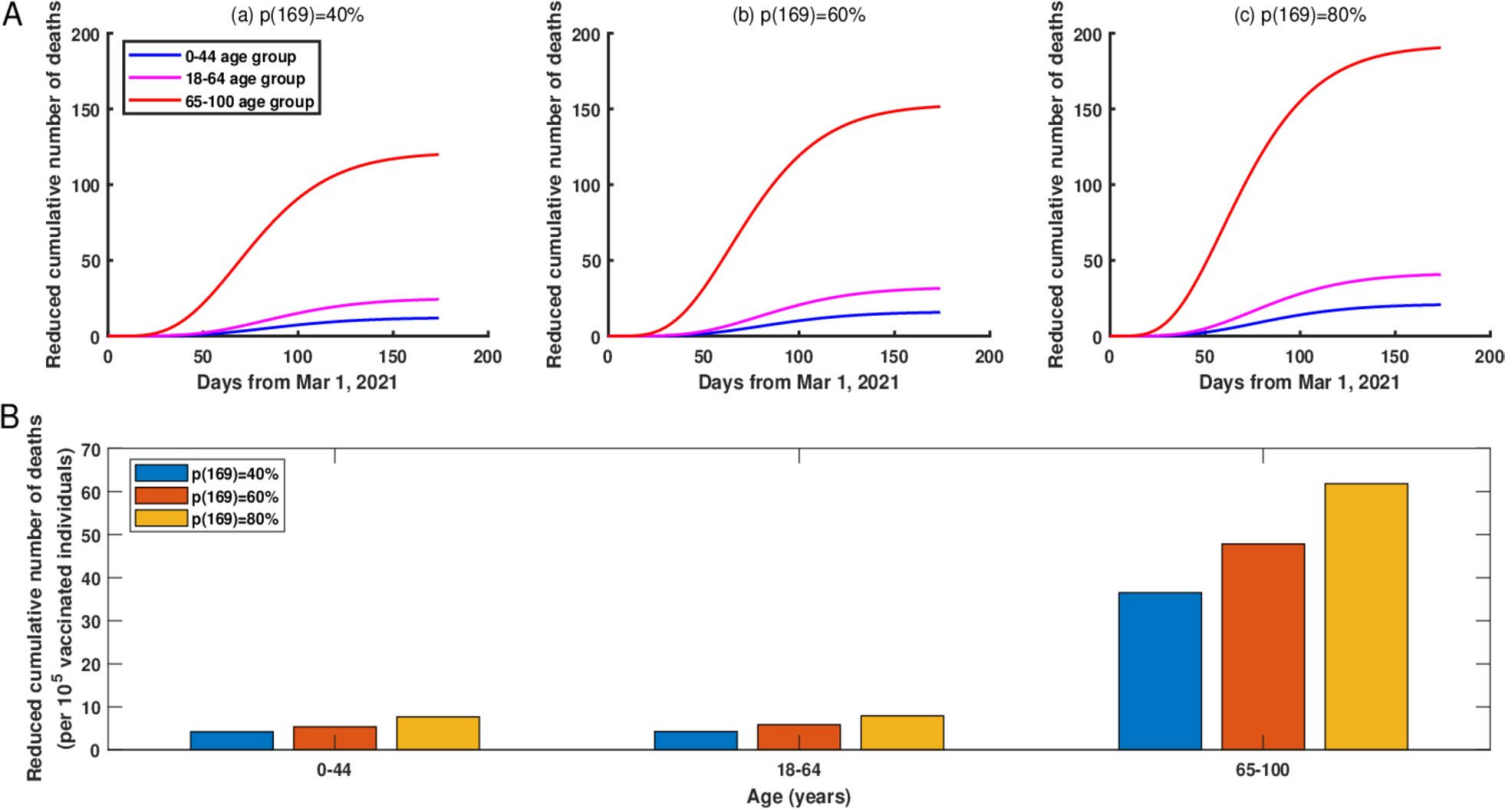
**A** The reduced cumulative number of deaths in NYC if starting from March 1, 2021, the vaccination coverage rates in 0–44, 18–64 and 65–100 age groups would gradually reach to 40%, 60%, and 80% by June 1, 2021, respectively. (a) The vaccination coverage rate was 40%. (b) The vaccination coverage rate was 60%. (c) The vaccination coverage rate was 80%. **B** The reduced cumulative number of deaths per increased 100,000 vaccinated individuals in 0–44, 18–64 and 65–100 age groups if starting from March 1, 2021, the vaccination coverage rate for 0–44, 18–64 and 65–100 age groups were gradually increased to 40%, 60%, and 80% by June 1, 2021, respectively

If we gradually increased the vaccination coverage rate for two age groups (0–17 and 18–44 age groups, 18–44 and 45–64 age groups, or 65–74 and 75–100 age groups, that is, 0–44 age group, 18–64 age group, or 65–100 age group) to 40% by June 1, 2021, then compared with the current vaccination strategy, the 65–100 age group would have the greatest reduction in cumulative deaths, about 83 fewer deaths as of June 1, 2021 (Figure 4A (a)). However, if the vaccination coverage rate in 0–44 age group, 18–64 age group, or 65–100 age group was increased to 60% by June 1, 2021, then, the 65–100 age group would still have the greatest reduction in cumulative deaths, about 110 fewer deaths as of June 1, 2021 (Figure 4A (b)). In general, the vaccination coverage rate was negative correlation with cumulative deaths.

In addition, similar with vaccination for only one age group, the increased vaccination coverage rate would lead to an obvious reduction in the cumulative number of deaths in two age groups, especially for the people at the age of 65–100 (Fig. 4B (a–c)). Specifically, if starting from March 1, 2021, the vaccination coverage rate in the 65–100 age group was gradually increased up to 40%, 60%, and 80% by June 1, 2021 in NYC, then through normalization, we obtained the reduced cumulative number of deaths were 36, 48, and 62 per increased 100,000 vaccinated individuals, respectively.

#### Reduction of cumulative new infections

**Fig. 5:**
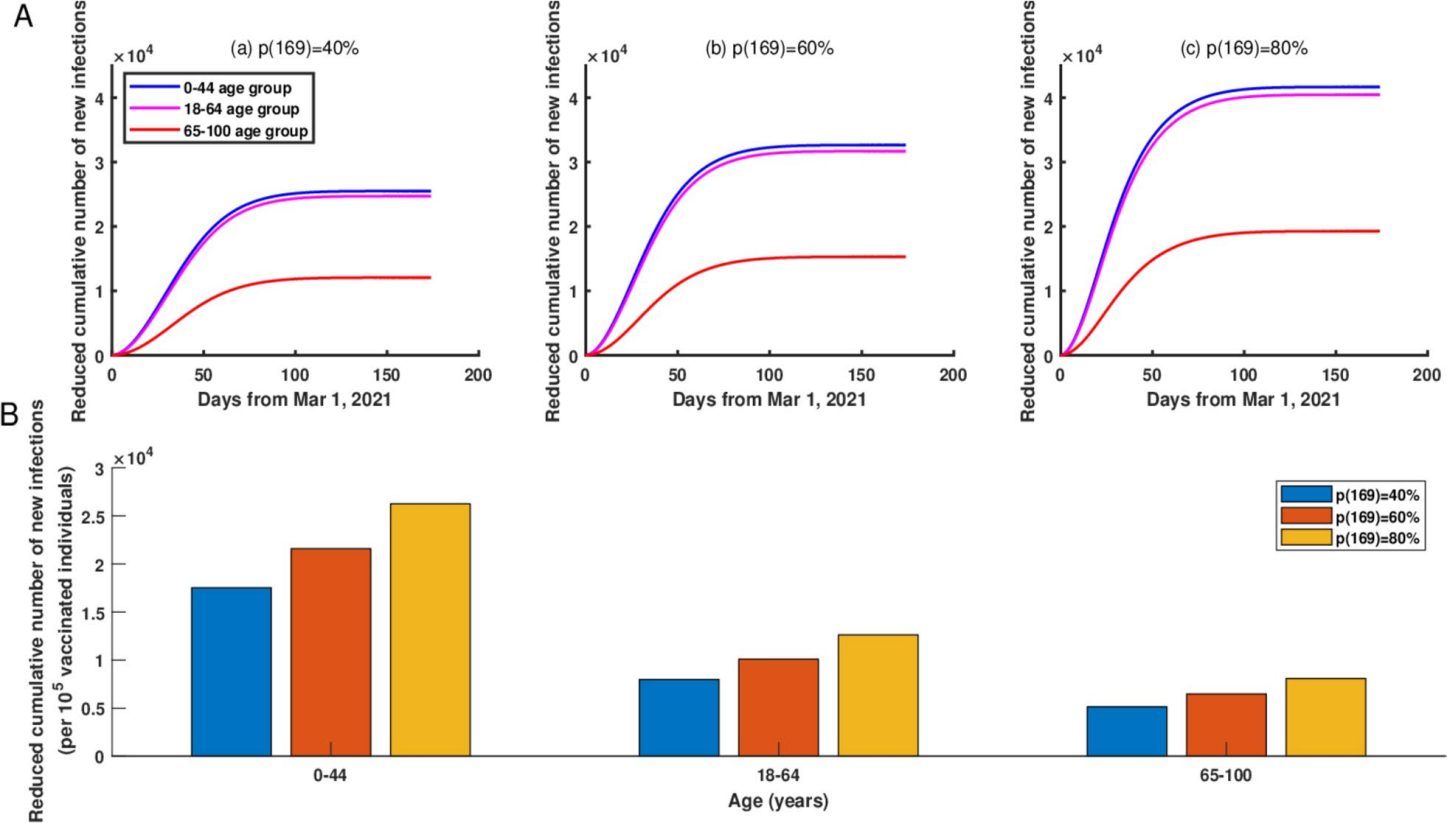
**A** The reduced cumulative number of new infections in NYC if starting from March 1, 2021, the vaccination coverage rates in 0–44, 18–64 and 65–100 age groups would gradually reach to 40%, 60%, and 80% by June 1, 2021, respectively. (a) The vaccination coverage rate was 40%. (b) The vaccination coverage rate was 60%. (c) The vaccination coverage rate was 80%. **B** The reduced cumulative number of new infections per increased 100,000 vaccinated individuals in the two groups if starting from March 1, 2021, the vaccination coverage rate for 0–44, 18–64 and 65–100 age groups were gradually increased to 40%, 60%, and 80% by June 1, 2021, respectively

If starting from March 1, 2021, we gradually increased the vaccination coverage rate for 0–44 age group, 18–64 age group, or 65–100 age group to 40% by June 1, 2021, then compared with the current vaccination strategy, the 0–44 age group would have the greatest reduction in cumulative new infections, about 24902 fewer new infections as of June 1, 2021 (Figure 5A (a)). In addition, if we gradually increased the vaccination coverage rate for 0–44 age group, 18–64 age group, or 65–100 age group to 60% by June 1, 2021, then the 0–44 age group would still have the greatest reduction in cumulative new infections, about 32034 fewer new infections as of June 1, 2021 (Figure 5A (b)). In general, the vaccination coverage rate was significantly negative correlation with the cumulative new infections in NYC (Figure 5A).

In addition, the increased vaccination coverage rate would lead to an obvious reduction in the cumulative number of new infections per increased 100,000 vaccinated individuals (Fig. 5B (a–c)). Specifically, if starting from March 1, 2021, the vaccination coverage rate in the 0–44 age group was gradually increased to 40%, 60%, and 80% by June 1, 2021, then through normalization, we obtained the reduced cumulative number of new infections were 17,515, 21,581 and 26,263 per increased 100,000 vaccinated individuals, respectively. Furthermore, we found that increasing the vaccination rate for younger age group would reduce the cumulative number of new infections even more in NYC.

### Impact of different vaccine allocation schemes

**Fig. 6:**
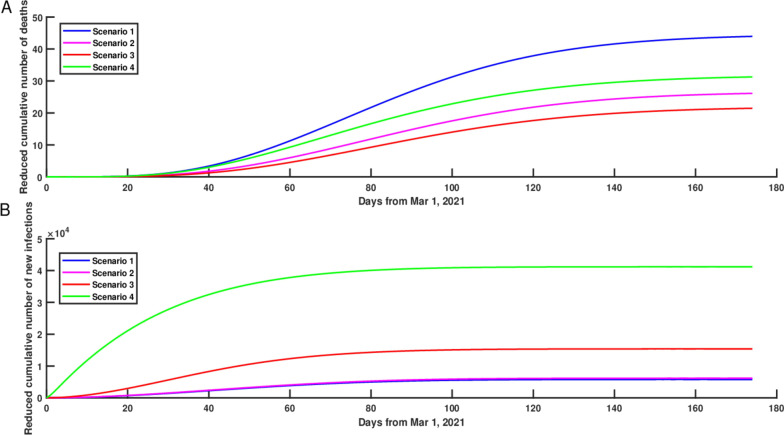
The reduced cumulative numbers of deaths (**A**) and new infections (**B**) in NYC if we reallocated vaccines to the 0–17 and 75–100 age groups starting from March 1, 2021, such that the proportions of vaccinated individuals in the 0–17 and 75–100 age groups on June 1, 2021 were reallocated according to the following four scenarios: (1) 0–17 age group 20% and 75–100 age group 80%; (2) 0–17 age group 40% and 75–100 age group 60%; (3) 0–17 age group 60% and 75–100 age group 40%; (4) 0–17 age group 80% and 75–100 age group 20%

**Fig. 7:**
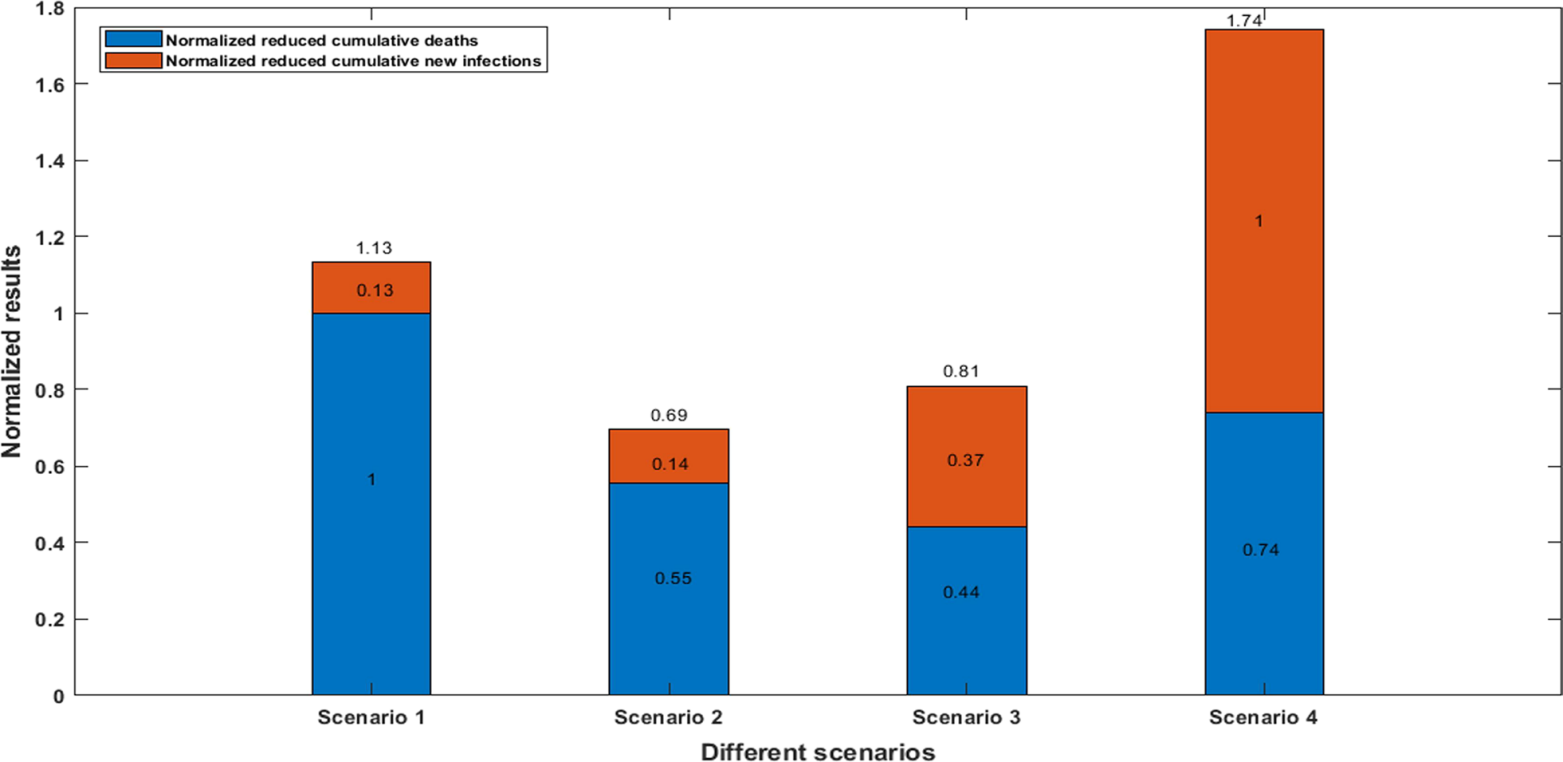
The normalized reduced cumulative numbers of deaths and new infections in NYC if we reallocated vaccines to the 0–17 and 75–100 age groups starting from March 1, 2021, such that the proportions of vaccinated individuals in the 0–17 and 75–100 age groups on June 1, 2021 were reallocated according to the following four scenarios: (1) 0–17 age group 20% and 75–100 age group 80%; (2) 0–17 age group 40% and 75–100 age group 60%; (3) 0–17 age group 60% and 75–100 age group 40%; (4) 0–17 age group 80% and 75–100 age group 20%

Based on the results of “Impact of vaccination for one age group” section, we found that if we gradually increased the vaccination coverage rate for only one age group (0–17 age group, 18–44 age group, 45–64 age group, 65–74 age group, or 75–100 age group) to 40%, 60% and 80% by June 1, 2021, then compared with the current vaccination strategy, the 0–17 age group would reduce the cumulative number of new infections mostly and the 75–100 age group would reduce the cumulative number of deaths mostly in NYC as of June 1, 2021. Therefore, 0–17 age group was considered as age group A, 75–100 age group was considered as age group B. Based on the current vaccination coverage rate of five age groups, we reallocated a batch of additional vaccines to these two age groups such that as of June 1, 2021, there would be additional 100,000 vaccinated individuals in the 0–17 age group and the 75–100 age group. We evaluated four vaccine allocation schemes as shown in “Impact of age-specific vaccination strategies” section third scenario, and calculated the reduced cumulative number of deaths and new infections in these two age groups. From Fig. 6A, we found that corresponding to four vaccine allocation schemes, compared with the current vaccination strategy, the reduced cumulative number of deaths were 28, 16, 12 and 21, respectively, and the corresponding reduced cumulative number of new infections were 5392, 5726, 14892 and 40688, respectively (Figure 6B). To compare the vaccine allocation scheme which would reduce the cumulative number of deaths and new infections most simultaneously, we mapped the reduced cumulative number of deaths in the four vaccine allocation schemes into the range of 0 to 1 through normalization, that is, the largest reduced cumulative number of deaths in the first allocation scheme was regarded as 1, and then the reduced cumulative number of deaths in the remaining three vaccine allocation schemes (scheme 2, scheme 3, and scheme 4) were scaled as 0.55, 0.44 and 0.74 respectively proportionally. Similarly, the largest reduced cumulative number of new infections in the fourth allocation scheme was regarded as 1, and then the reduced cumulative number of new infections in the remaining three vaccine allocation schemes (scheme 1, scheme 2, and scheme 3) were scaled as 0.13, 0.14 and 0.37 respectively proportionally. Adding the normalized results for the reduced cumulative number of deaths and new infections in each vaccine allocation scheme, then we obtained the final normalized results of the four vaccine allocation schemes (scheme 1, scheme 2, scheme 3 and scheme 4) were 1.13, 0.69, 0.81 and 1.74 respectively. Therefore, we obtained the fourth vaccine allocation scheme (0–17 age group accounted for 80% and 75–100 age group accounted for 20%) was the most optimal, the reduced cumulative numbers of deaths and new infections were 21 and 40,688 per increased 100,000 vaccinated individuals, respectively, which could ensure the greatest reduction in the cumulative number of deaths and new infections simultaneously in NYC (Figure 7).

## Discussion

The COVID-19 pandemic has devastated families and societies in health and economy around the world including NYC. Although social distancing, quarantine, isolation and lockdown restrictions were effective in limiting the infection and spread of SARS-CoV-2 in a short term, but the absence of immunity in the population leave them susceptible to further waves of SARS-CoV-2 infection. Effective and safe vaccines, when available, would most likely become our best tool for the global epidemic control of COVID-19. However, vaccine production is likely insufficient at present, even in developed and high income areas such as NYC [12, 13]. NYC has started the first batch of SARS-Cov-2 vaccines since December 14, 2020. However, the initial vaccination rate was slow and the supply of COVID-19 vaccines was insufficient. Therefore, optimizing age-specific vaccination strategies is currently an important issue in NYC and other countries.

In this study, combined with three different types of reported COVID-19 data from March 24, 2020 to February 28, 2021 in NYC, we established a mathematical model with 5 age groups according to the transmission characteristics of SARS-CoV-2, and estimated the most optimal vaccine allocation strategies. Our results showed that if we increased the vaccination rate to 40% in each age group on June 1, 2021, the reduced total number of cumulative deaths in the 75–100, 65–74 and 45–64 age groups were 63, 23 and 10 as of June 1, 2021, respectively. And the reduced number of new infections in the 18–44, 0–17 and 45–64 age groups were 13,463, 12,807 and 11,073, respectively. More importantly, if we distributed a batch of additional vaccines to the 0–17 and 75–100 age group such that there would be additional 100,000 vaccinated individuals in these two age groups as of June 1, 2021, by comparing the impact of different vaccine allocation schemes, we found that allocating these vaccines such that 0–17 age group accounted for 80% and 75–100 age group accounted for 20% was the most optimal and could ensure the greatest reduction in the cumulative number of deaths and new infections simultaneously in NYC. These results especially emphasize the importance of children's vaccination to control the epidemic [33–35].

As expected, our results showed that the COVID-19 burden including deaths and new infections, decreased with increasing vaccination rate. Our model showed that rapid increase in the vaccination rate was necessary to achieve significant reduction in disease burden. Furthermore, we used mathematical optimization to determine the vaccine allocation by age-specific vaccination, and did not impose any restrictions in the allocation strategies. When vaccines are fully available, a feasible solution could allocate vaccine to the high-risk groups and the high-transmission groups firstly, such as elderly and children.

The study has several strengths. First, we considered the exposed and asymptomatic individuals in our age-structured mathematical model, which was more in line with the actual process of COVID-19 infection. Secondly, we considered the different contact rates between 5 age groups in different periods, which was more in line with the actual prevention and control for the COVID-19 pandemic in NYC. Finally, we considered the vaccinated individuals in our age-structured mathematical model and evaluated the impact of different vaccination strategies on the cumulative number of deaths and new infections of COVID-19.

On the other hand, our study also has several limitations. First, we considered that the entire population of NYC was homogeneous and ignored the heterogeneity of population distribution. Secondly, we assumed that the contact matrix in each period was symmetric, and assumed that the contact rates of 5 age groups made by the 75–100 age group were equal. Thirdly, we assumed that the recovered individuals were assumed to be complete immune to the COVID-19 and would not be reinfected. However, the immunity from previous infection is only protective against 80–91% reinfection [36–39]. Again, the vaccination protective against reinfection of new VOC Omicron could be lower [40]. Fourthly, we have not considered the effect of several factors such as societal, ethical and political factors on the implementation of optimal strategies. Besides, we have not considered the comparison of predictions and actual data starting from March 1, 2021 for simplicity. In addition, our model has not combined the effect of implementing the routine vaccination program with social distancing and face mask usage. This study also did not consider to what extent age-specific epidemiological parameters depend on the absolute numbers and degree of social distancing in the different age groups in our mathematical model. Furthermore, there is no clear evidence to draw definite conclusions about the characteristics of the new SARS-CoV-2 variants, for simplicity, the emergence of new variants has not been taken into consideration in this study. Studies have shown that the third dose of booster vaccination can further increase the protection, however, we did not consider the effect of the booster dose in this study. Finally, we did not consider the deaths of asymptomatic infections and symptomatic individuals who were not hospitalized, despite the very low mortality in these population. Due to these limitations, this study can only make inferences about the results obtained.

## Conclusions

Efficient and safe vaccines are considered to be the best tool to control the COVID-19 pandemic. In the early days of vaccine supply, vaccine shortage was an inevitable problem. In this study, we used a mathematical model with five age groups to estimate the effect of age-specific vaccine allocation strategies on reducing the cumulative number of deaths and new infections. We found that priority vaccination to the youngest age group would reduce the new infections most, and priority vaccination to the middle and elderly age groups would reduce the deaths most. Besides, priority vaccination to the elderly and adolescents would minimize both the deaths and the new infections. In addition, the effect of vaccine allocation was cumulative when additional vaccinations were allocated to more than one age group. Our results in this study can be used by public health physicians and policy decision makers in NYC as well as other countries and regions.

## Supplementary Information

**Additional file 1. Text S1:** Model formulation.

**Additional file 2. Figures:** Figure S1. Model calibration with the reported cumulative number of confirmed cases. (a) In the all age groups of NYC. (b) In 0-17 age group. (c) In 18-44 age group. (d) In 45-64 age group. (e) In 65-74 age group. (f) In 75-100 age group. Figure S2. Model calibration with the reported cumulative number of deaths. (a) In the all age groups of NYC. (b) In 0-17 age group. (c) In 18-44 age group. (d) In 45-64 age group. (e) In 65-74 age group. (f) In 75-100 age group. Figure S3. Model calibration with the reported cumulative number of hospitalizations. (a) In the all age groups of NYC. (b) In 0-17 age group. (c) In 18-44 age group. (d) In 45-64 age group. (e) In 65-74 age group. (f) In 75-100 age group.

**Additional file 3. Tables:** Table S1. The reported COVID-19 data in 5 age groups (0-17, 18-44, 45-64, 65-74 and 75-100) in NYC from the official website of New York from March 24, 2020 to February 28, 2021. Columns 2-6. cumulative confirmed cases, Columns 7-11. cumulative deaths, Columns 12-16. cumulative hospitalizations. Table S2. Parameter description and estimated values for models (1) and (2) without vaccination. All the unknown initial values and parameters as well as their 95% confidence intervals were estimated by using the Markov Chain Monte Carlo (MCMC) approach. Table S3. Parameter description and estimated values for models (3) and (4) with vaccination. All the unknown parameters as well as their 95% confidence intervals were estimated by using the Markov Chain Monte Carlo (MCMC) approach.

## Data Availability

Data and Methods are available by contacting the corresponding authors.

## Abbreviations

NYC: New York City
VOI: Variants of interest
VOC: Variants of concern
MCMC: Markov Chain Monte Carlo
SARS-CoV-2: Severe acute respiratory syndrome coronavirus 2
SVEAICHR: Susceptible-vaccinated-exposed-asymptomatic-symptomatic-confirmed-hospitalized-recovered
