# Supplementary material for: COVID-19 epidemic in New York City: development of an age group-specific mathematical model to predict the outcome of various vaccination strategies
Source: Virol J. 2022 Mar 15;19:43. doi: 10.1186/s12985-022-01771-9 (PMC8922400; doi:10.1186/s12985-022-01771-9)
Supplement: Supplementary file 1 — Additional file 1. Text S1: Model formulation. [file 12985_2022_1771_MOESM1_ESM.docx]

**Supplementary material for model formulation**

1. **Model formulation**

According to the natural history of COVID-19 and the actual prevention and control for the COVID-19 pandemic in NYC, we established a mathematical model with 5 age groups at the population level by considering exposed and asymptomatic infections [1-3]. To simplify, we assumed that the maximum age of people was 100 years old, and the total population in NYC were divided into 5 age groups (0-17, 18-44, 45-64, 65-74 and 75-100 years) [4]. The sub-population in each age group $i$ in NYC were further divided into eight compartments: susceptible individuals$S_{i}(t)$; vaccinated individuals $V_{i}(t)$; exposed individuals$E_{i}(t)$; infected but asymptomatic individuals $A_{i}(t)$; infected and symptomatic individuals$I_{i}(t)$; confirmed cases who stayed at home$C_{i}(t)$; hospitalized individuals $H_{i}(t)$ and recovered individuals$R_{i}(t)$.

For the sake of simplicity, we made the following assumptions:

1. The entire population of NYC was considered to be homogeneous and the heterogeneity of population distribution was not considered.
2. The contact rate of the age group $i$ made by age group $j$ was assumed to be equal to the contact rate of the age group $j$ made by age group $i$.
3. The contact rates of the 75-100 age group made by other age groups were assumed to be equal.
4. The death rates of asymptomatic individuals, symptomatic individuals and confirmed individuals (not hospitalized) were not considered.
5. The recovered individuals were assumed to be immune to the COVID-19 and could avoid the re-infection.
   1. **Model without vaccination**

According to the above assumptions, the age-structured mathematical model at the population level without considering vaccine was described by the following equations (1) and (2).

$\begin{matrix} dS_{i}/dt=-\Lambda S_{i}-p(t)S_{i}, \\ dE_{i}/dt=\Lambda S_{i}-\varepsilon E_{i}, \\ dA_{i}/dt=(1-\eta_{i})\varepsilon E_{i}-\gamma_{1}A_{i}, \\ dI_{i}/dt=\eta_{i}\varepsilon E_{i}-\delta_{i}I_{i}, \\ dC_{i}/dt=\delta_{i}I_{i}-(\alpha_{i}(t)+\gamma_{2})C_{i}, \\ dH_{i}/dt=\alpha_{i}(t)C_{i}-(\mu_{i}(t)+\gamma_{3})H_{i}, \\ dR_{i}/dt=\gamma_{1}A_{i}+\gamma_{2}C_{i}+\gamma_{3}H_{i}. \end{matrix}$ (1)

where $S(t)=\sum_{i=1}^{5} S_{i}(t)$, $I(t)=\sum_{i=1}^{5} I_{i}(t)$, $C(t)=\sum_{i=1}^{5} C_{i}(t)$, $E(t)=\sum_{i=1}^{5} E_{i}(t)$, $A(t)=\sum_{i=1}^{5} A_{i}(t)$, $R(t)=\sum_{i=1}^{5} R_{i}(t)$, $N(t)=S(t)+E(t)+A(t)+I(t)+R(t)$.

Here, the force of infection in equation (1) was described as:

$\Lambda=\beta\sum_{j=1}^{5} c_{ij}(t)\frac{(I_{j}(t)+\theta_{1}E_{j}(t)+\theta_{2}A_{j}(t)+\theta_{3}C_{j}(t))}{N_{j}(t)}$,

where $c_{ij}(t)$ was the contact rate of age group $i$ made by age group $j$ at time $t$.

Besides, we assumed that when more healthcare resources became available, the death rate of hospitalized individuals and the transfer rate from confirmed cases to hospitalized individuals were exponentially decreasing functions:

$\mu_{i}(t)=\mu_{1i}+\mu_{2i}\exp(-\mu_{3i}t)$, $\alpha_{i}(t)=\alpha_{1i}+\alpha_{2i}\exp(-\alpha_{3i}t)$.

The cumulative number of confirmed cases $Y_{i}$ in age group $i$, the cumulative number of deaths $Z_{i}$in age group $i$ and the cumulative number of hospitalizations$P_{i}$ in age group $i$ were given by:

$\begin{matrix} dY_{i}/dt=\delta_{i}I_{i}(t), \\ dZ_{i}/dt=\mu_{i}(t)H_{i}(t), \\ dP_{i}/dt=\alpha_{i}(t)C_{i}(t). \end{matrix}$ (2)

- 1. **Model with vaccination**

When the vaccine for COVID-19 became available, the susceptible individuals$S_{i}(t)$ would be vaccinated and became the vaccinated individuals $V_{i}(t)$. The vaccination coverage rate $p(t)$ was assumed to be a logistic function, that is, $p(t)=\frac{p_{\max}p_{0}}{p_{0}-(p_{0}-p_{\max})\exp(-rt)}$, where $p_{\max}$was the maximum vaccination coverage rate, $p_{0}$was the initial vaccination coverage rate and $r$ was the vaccination rate in NYC. The effectiveness of the vaccine for COVID-19 $q$ was assumed to be 0.95 [5]. The vaccinated individuals $V_{i}(t)$ would be infected by contacts with infected and symptomatic individuals $I_{i}(t)$, exposed individuals$E_{i}(t)$, infected but asymptomatic individuals $A_{i}(t)$ and confirmed cases$C_{i}(t)$. Therefore, the age-structured mathematical model at the population level with considering vaccine was described by the following equations (3) and (4).

$\begin{matrix} dS_{i}/dt=-\Lambda S_{i}-p(t)S_{i}, \\ dV_{i}/dt=p(t)S_{i}-(1-q)\Lambda V_{i}, \\ dE_{i}/dt=\Lambda S_{i}+(1-q)\Lambda V_{i}-\varepsilon E_{i}, \\ dA_{i}/dt=(1-\eta_{i})\varepsilon E_{i}-\gamma_{1}A_{i}, \\ dI_{i}/dt=\eta_{i}\varepsilon E_{i}-\delta_{i}I_{i}, \\ dC_{i}/dt=\delta_{i}I_{i}-(\alpha_{i}(t)+\gamma_{2})C_{i}, \\ dH_{i}/dt=\alpha_{i}(t)C_{i}-(\mu_{i}(t)+\gamma_{3})H_{i}, \\ dR_{i}/dt=\gamma_{1}A_{i}+\gamma_{2}C_{i}+\gamma_{3}H_{i}. \end{matrix}$ (3)

where $S(t)=\sum_{i=1}^{5} S_{i}(t)$, $I(t)=\sum_{i=1}^{5} I_{i}(t)$, $C(t)=\sum_{i=1}^{5} C_{i}(t)$, $V(t)=\sum_{i=1}^{5} V_{i}(t)$, $E(t)=\sum_{i=1}^{5} E_{i}(t)$, $A(t)=\sum_{i=1}^{5} A_{i}(t)$, $R(t)=\sum_{i=1}^{5} R_{i}(t)$,

$N(t)=S(t)+V(t)+E(t)+A(t)+I(t)+R(t)$.

Here, the force of infection in equation (3) was described as:

$\Lambda=\beta\sum_{j=1}^{5} c_{ij}(t)\frac{(I_{j}(t)+\theta_{1}E_{j}(t)+\theta_{2}A_{j}(t)+\theta_{3}C_{j}(t))}{N_{j}(t)}$,

where $c_{ij}(t)$ was the contact rate of age group $i$ made by age group $j$ at time $t$.

In addition, we still assumed that when more healthcare resources became available, the death rate of hospitalized individuals and the transfer rate from confirmed cases to hospitalized individuals were exponentially decreasing functions:

$\mu_{i}(t)=\mu_{4i}+\mu_{5i}\exp(-\mu_{6i}t)$, $\alpha_{i}(t)=\alpha_{4i}+\alpha_{5i}\exp(-\alpha_{6i}t)$.

The cumulative number of confirmed cases $Y_{i}$ in age group $i$, the cumulative number of deaths $Z_{i}$in age group $i$ and the cumulative number of hospitalizations$P_{i}$ in age group $i$ were given by:

$\begin{matrix} dY_{i}/dt=\delta_{i}I_{i}(t), \\ dZ_{i}/dt=\mu_{i}(t)H_{i}(t), \\ dP_{i}/dt=\alpha_{i}(t)C_{i}(t). \end{matrix}$ (4)

**1.3 Initial conditions of model (1) and (2) and input parameters**

We collected three different types of reported COVID-19 data in 5 age groups (0-17, 18-44, 45-64, 65-74 and 75-100) in NYC from the official website of New York [6, 7], specifically including daily reported cumulative confirmed cases **(Supplementary table 1, columns 2-6)**, daily reported cumulative deaths **(Supplementary table 1, columns 7-11)** and daily reported cumulative hospitalizations **(Supplementary table 1, columns 12-16)** from March 24, 2020 to December 13, 2020. Therefore, March 24, 2020 was chosen as the initial time for model (1) and (2).

The entire population of NYC was 8,398,748 [8]. The total population at the initial time $N_{i}(0)$ in each age group $i$ in NYC was calculated based on the age-specific proportion of the population in 2017 [9]. Since there was no recovered individual in NYC as of March 24, 2020, so we assumed that the initial values of recovered individuals $R_{i}(0)=0$. For simplicity, we assumed that the initial values of confirmed cases $C_{i}(0)=Y_{i}(0)-P_{i}(0)-R_{i}(0)$. Similarly, we assumed that the initial values of the existing hospitalizations $H_{i}(0)=P_{i}(0)-R_{i}(0)$. The initial values of the exposed individuals$E_{i}(0)$, infected but asymptomatic individuals $A_{i}(0)$, and infected and symptomatic individuals$I_{i}(0)$ were estimated with the reported data. Then we could obtain the initial values of the susceptible individuals $S_{i}(0)=N_{i}(0)-E_{i}(0)-A_{i}(0)-I_{i}(0)-C_{i}(0)-H_{i}(0)-R_{i}(0)$. The initial values of the cumulative confirmed cases$Y_{i}(0)$, cumulative deaths $Z_{i}(0)$, and cumulative hospitalizations $P_{i}(0)$ were obtained based on the reported data on March 24, 2020.

Since NYC had implemented stay-at-home orders from March 23, 2020 and reopened from June 8, 2020. In particular, NYC had restarted offline teaching from September 21, 2020 [10]. Therefore, we assumed that the contact rate of age group $i$ made by age group $j$ was $c_{ij}$ from March 24, 2020 to June 7, 2020. Reopening and offline teaching would have resulted in an increase in the contact rate in NYC. Here, for simplicity, we assumed that the contact rate of the age group $i$ made by age group $j$ was $(1+k_{1})c_{ij}$ from June 8, 2020 to September 20, 2020, and the contact rate of the age group $i$ made by age group $j$ was $(1+k_{2})c_{ij}$ from September 21, 2020 to December 13, 2020. The other unknown parameters in the models (1) and (2) were estimated with the reported data in NYC by using the Markov Chain Monte Carlo (MCMC) approach. Specially, the basic steps of MCMC algorithm as follows: Step 1. Collect sample observations; Step 2. Determine the conditional density function; Step 3. Construct the likelihood function; Step 4. Establish the probability of joint posterior distribution; Step 5. Select the initial value of parameters; Step 6. Determine the acceptance probability; Step 7. Select the random number from the Uniform distribution U (0,1)，and determine whether accept or reject the candidate parameter; Step 8. Select a burn-in period of m times and a cycle of N times, calculate the mean value of the last N-m times about the estimated parameters and take them as the estimated values. We set the iteration number to 8,000 and the burn-in periods to 7,500. By using the Markov Chain Monte Carlo (MCMC) approach, we obtained the unknown initial values and parameters in the models (1) and (2). **Supplementary table 2** described the estimated initial values and parameters as well as their 95% confidence intervals.

**1.4 Initial conditions of model (3) and (4) and input parameters**

On December 14, 2020, NYC began to vaccinate the first batch of COVID-19 vaccines. We obtained the reported COVID-19 data in 5 age groups (0-17, 18-44, 45-64, 65-74 and 75-100) in NYC from the official website of New York [6, 7], specifically including daily reported cumulative confirmed cases **(Supplementary table 1, columns 2-6)**, daily reported cumulative deaths **(Supplementary table 1, columns 7-11)** and daily reported cumulative hospitalizations **(Supplementary table 1, columns 12-16)** from December 14, 2020 to February 28, 2021. Therefore, December 14, 2020 was chosen as the initial time for models (3) and (4).

On December 14, 2020, approximately 10,000 people in New York State were vaccinated with COVID-19 vaccines [11]. Since the total population in New York State was 19.54 million [12], so the initial vaccination coverage rate in New York State on December 14, 2020 was and we used as the initial vaccination coverage rate in NYC. Besides, we assumed that the maximum vaccination coverage $p_{\max}$was 95% in NYC. The effectiveness of the COVID-19 vaccines $q$ was assumed to be 95% [5].

Based on the initial values and parameters in the models (1) and (2), we calculated the exposed individuals; infected but asymptomatic individuals; infected and symptomatic individuals; confirmed cases; hospitalized cases and recovered cases on December 14, 2020 in the model (1) , and we used them as the initial values of exposed individuals; infected but asymptomatic individuals; infected and symptomatic individuals; confirmed cases; hospitalized cases and recovered cases on December 14, 2020 in the model (3). Similarly, we calculated the susceptible individuals on December 14, 2020 in the model (1) . For simplicity, we assumed that the initial values of the vaccinated individuals $V_{i}(0)$ were equal to the susceptible individuals on December 14, 2020 in the model (1) multiplied by the initial vaccination coverage rate $p_{0}$, and the initial values of the susceptible individuals$S_{i}(0)$ were equal to the susceptible individuals on December 14, 2020 in the model (1) multiplied by $(1-p_{0})$. The initial values of the cumulative confirmed cases$Y_{i}(0)$, cumulative deaths $Z_{i}(0)$, and cumulative hospitalizations $P_{i}(0)$ were obtained from the reported COVID-19 data on December 14, 2020 in NYC.

Since the Christmas break in NYC was from December 24, 2020 to January 1, 2021, here we assumed that the contact rate in NYC may have changed one week before and after the Christmas break. We assumed that the contact rate of the age group $i$ made by age group $j$ was $k_{ij}$ from December 17, 2020 to January 8, 2021, the contact rate of the age group $i$ made by age group $j$ was $m_{ij}$ from January 9, 2021 to February 28, 2021, the contact rate of the age group $i$ made by age group $j$ was $(1+k_{2})c_{ij}$ from December 14, 2020 to December 16, 2021.

Vaccination may change the hospitalization rate, the death rate of hospitalized cases. Here, we assumed that the hospitalization rate $\alpha_{i}(t)=\alpha_{4i}+\alpha_{5i}\exp(-\alpha_{6i}t)$ where $\alpha_{5i}$ was the hospitalization rate on December 14, 2020 and could be calculated based on the initial values and parameters in the models (1) and (2). The death rate of hospitalized cases was assumed that $\mu_{i}(t)=\mu_{4i}+\mu_{5i}\exp(-\mu_{6i}t)$ where $\mu_{5i}$ was the death rate of hospitalized cases on December 14, 2020 and could also be calculated based on the initial values and parameters in the models (1) and (2). Besides, the transfer rate from symptomatic infections to confirmed cases $\delta_{i}$ and the proportion of symptomatic infections $\eta_{i}$ may change, so we re-estimated the transfer rate from symptomatic infections to confirmed cases $\delta_{i}$ the proportion of symptomatic infections $\eta_{i}$. The other parameters in the models (3) and (4) were assumed to remain unchanged. By using the Markov Chain Monte Carlo (MCMC) approach, we obtained the estimated parameters in the models (3) and (4). The description of estimated parameters as well as their 95% confidence intervals in the models (3) and (4) were described in **Supplementary Table 3**.

**References**

1. Zhu, Y, Chen, Y.Q. On a statistical transmission model in analysis of the early phase of COVID-19 outbreak. Statistics in biosciences. 2021, 13(1): 1-17.
2. Kasraeian, M, Zare, M, Vafaei, H, et al. COVID-19 pneumonia and pregnancy; a systematic review and meta-analysis. The Journal of Maternal-Fetal & Neonatal Medicine. 2020, 1-8.
3. Wilder, B, Charpignon, M, Killian, JA, et al. Modeling between-population variation in COVID-19 dynamics in Hubei, Lombardy, and NYC. Proceedings of the National Academy of Sciences. 2020, 117(41): 25904-25910.
4. NYC Coronavirus Disease 2019 (COVID-19) Data. [https://github.com/nychealth/coronavirus-data](https://github.com/nychealth/coronavirus-data.)
5. US FDA staff backs Pfizer’s coronavirus vaccine (Update). https://cyprus-mail.com/2020/12/08/us-fda-staff-backs-pfizers-coronavirus-vaccine/
6. NYC Health. 2020. COVID-19 Data. [https://www1.nyc.gov/site/doh/covid/covid-19-data-archive.page](https://www1.nyc.gov/site/doh/covid/covid-19-data-archive.page.)
7. NYC Coronavirus Disease 2019 (COVID-19) Data. [https://github.com/nychealth/coronavirus-data](https://github.com/nychealth/coronavirus-data.)
8. [NYCdata: Metropolitan Statistical Area (MSA) Population](https://www.baruch.cuny.edu/nycdata/population-geography/population.htm). [https://www.baruch.cuny.edu/nycdata/population-geography/population.htm](https://www.baruch.cuny.edu/nycdata/population-geography/population.htm.)
9. NYC (NYC) Age and Sex Distribution-By County. [https://www.baruch.cuny.edu/nycdata/population-geography/age_distribution.htm](https://www.baruch.cuny.edu/nycdata/population-geography/age_distribution.htm.)
10. NYC mayor says first day of in-person classes delayed until September 21.https://www.cbsnews.com/news/nyc-schools-reopening-delayed-september-21-de-blasio/
11. Nearly 10,000 doctors in New York have been vaccinated against the new crown?! [https://www.sohu.com/a/438483677_642002](https://www.sohu.com/a/438483677_642002.) (in Chinese)
12. Economic and Commercial Office of the Consulate General of the People's Republic of China in New York. [https://baike.baidu.com/reference/3439049/3007VaFuklR2s13x0RF5e5DR6zFv9dskU_aEtjP8N3cNzPHL4mJgtAMO191DvHj1T1Aj0ziGcSALgk34cml6Br2UDjqvbSdmN5o7AVEaOwlPt0V-vQuh3FmQfozUAvGdHiRfbDkYbg](https://baike.baidu.com/reference/3439049/3007VaFuklR2s13x0RF5e5DR6zFv9dskU_aEtjP8N3cNzPHL4mJgtAMO191DvHj1T1Aj0ziGcSALgk34cml6Br2UDjqvbSdmN5o7AVEaOwlPt0V-vQuh3FmQfozUAvGdHiRfbDkYbg.)
