# Supplementary material for: COVID-19 epidemic in New York City: development of an age group-specific mathematical model to predict the outcome of various vaccination strategies
Source: Virol J. 2022 Mar 15;19:43. doi: 10.1186/s12985-022-01771-9 (PMC8922400; doi:10.1186/s12985-022-01771-9)
Supplement: Supplementary file 2 — Additional file 2. Figures: Figure S1. Model calibration with the reported cumulative number of confirmed cases. (a) In the all age groups of NYC. (b) In 0-17 age group. (c) In 18-44 age group. (d) In 45-64 age group. (e) In 65-74 age group. (f) In 75-100 age group. Figure S2. Model calibration with the reported cumulative number of deaths. (a) In the all age groups of NYC. (b) In 0-17 age group. (c) In 18-44 age group. (d) In 45-64 age group. (e) In 65-74 age group. (f) In 75-100 age group. Figure S3. Model calibration with the reported cumulative number of hospitalizations. (a) In the all age groups of NYC. (b) In 0-17 age group. (c) In 18-44 age group. (d) In 45-64 age group. (e) In 65-74 age group. (f) In 75-100 age group. [file 12985_2022_1771_MOESM2_ESM.docx]

**Supplementary Table 2 Parameter description and estimated values for models (1) and (2) without vaccination.**

| Parameters | Meanings | Values | 95% CI | Estimation method |
| --- | --- | --- | --- | --- |
| $\beta$ | Per-capita transmission rate | 0.0070 | (0.0069, 0.0070) | MCMC |
| $c_{11}$ | Contact rate of the age group 0-17 years made  by age group 0-17 years | 2.0399 | (2.0398, 2.0400) | MCMC |
| $c_{12}$ | Contact rate of the age group 18-44 years made  by age group 0-17 years | 3.2757 | (3.2757, 3.2758) | MCMC |
| $c_{13}$ | Contact rate of the age group 45-64 years made  by age group 0-17 years | 1.2243 | (1.2243, 1.2244) | MCMC |
| $c_{14}$ | Contact rate of the age group 65-74 years made  by age group 0-17 years | 0.5352 | (0.5351, 0.5352) | MCMC |
| $c_{15}$ | Contact rate of the age group 75-100 years made by age group 0-17 years | 0.3600 | (0.3600, 0.3601) | MCMC |
| $c_{22}$ | Contact rate of the age group 18-44 years made by age group 18-44 years | 3.1553 | (3.1553, 3.1554) | MCMC |
| $c_{23}$ | Contact rate of the age group 45-64 years made by age group 18-44 years | 2.1969 | (2.1969, 2.1970) | MCMC |
| $c_{24}$ | Contact rate of the age group 65-74 years made by age group 18-44 years | 0.6082 | (0.6081, 0.6083) | MCMC |
| $c_{33}$ | Contact rate of the age group 45-64 years made by age group 45-64 years | 0.5058 | (0.5057, 0.5058) | MCMC |
| $c_{34}$ | Contact rate of the age group 65-74 years made by age group 45-64 years | 0.8681 | (0.8680, 0.8681) | MCMC |
| $c_{44}$ | Contact rate of the age group 65-74 years made by age group 65-74 years | 0.4703 | (0.4703, 0.4704) | MCMC |
| $\eta_{1}$ | The proportion of symptomatic infections in the age group 0-17 years | 0.0262 | (0.0262, 0.0263) | MCMC |
| $\eta_{2}$ | The proportion of symptomatic infections in the age group 18-44 years | 0.0573 | (0.0573, 0.0574) | MCMC |
| $\eta_{3}$ | The proportion of symptomatic infections in the age group 45-64 years | 0.0893 | (0.0892, 0.0894) | MCMC |
| $\eta_{4}$ | The proportion of symptomatic infections in the age group 65-74 years | 0.1402 | (0.1402, 0.1403) | MCMC |
| $\eta_{5}$ | The proportion of symptomatic infections in the age group 75-100 years | 0.2298 | (0.2297, 0.2299) | MCMC |
| $\delta_{1}$ | Transfer rate from symptomatic individuals to confirmed cases in the age group 0-17 years | 0.0901 | (0.0900, 0.0901) | MCMC |
| $\delta_{2}$ | Transfer rate from symptomatic individuals to confirmed cases in the age group 18-44 years | 0.0609 | (0.0608, 0.0609) | MCMC |
| $\delta_{3}$ | Transfer rate from symptomatic individuals to confirmed cases in the age group 45-64 years | 0.0701 | (0.0700, 0.0702) | MCMC |
| $\delta_{4}$ | Transfer rate from symptomatic individuals to confirmed cases in the age group 65-74 years | 0.0862 | (0.0861, 0.0864) | MCMC |
| $\delta_{5}$ | Transfer rate from symptomatic individuals to confirmed cases in the age group 75-100 years | 0.8296 | (0.8286, 0.8306) | MCMC |
| $\alpha_{1}(t)$ | Transfer rate from confirmed cases to hospitalized cases in the age group 0-17 years | $\alpha_{1}(t)=\alpha_{11}+\alpha_{21}\exp(-\alpha_{31}t)$ | | MCMC |
|  | $\alpha_{11}$Minimum transfer rate from confirmed cases to hospitalized cases | 8.8197e-04 | (0.8481e-03, 0.9158e-03) |  |
|  | $\alpha_{21}$Initial value of transfer rate from confirmed cases to hospitalized cases | 0.0087 | (0.0087, 0.0088) |  |
|  | $\alpha_{31}$Exponential decline rate | 0.0099 | (0.0099, 0.0100) |  |
| $\alpha_{2}(t)$ | Transfer rate from confirmed cases to hospitalized cases in the age group 18-44 years | $\alpha_{2}(t)=\alpha_{12}+\alpha_{22}\exp(-\alpha_{32}t)$ | | MCMC |
|  | $\alpha_{12}$Minimum transfer rate from confirmed cases to hospitalized cases | 0.0032 | (0.0031, 0.0032) |  |
|  | $\alpha_{22}$Initial value of transfer rate from confirmed cases to hospitalized cases | 0.0082 | (0.0082, 0.0083) |  |
|  | $\alpha_{32}$Exponential decline rate | 0.0139 | (0.0138, 0.0139) |  |
| $\alpha_{3}(t)$ | Transfer rate from confirmed cases to hospitalized cases in the age group 45-64 years | $\alpha_{3}(t)=\alpha_{13}+\alpha_{23}\exp(-\alpha_{33}t)$ | | MCMC |
|  | $\alpha_{13}$Minimum transfer rate from confirmed cases to hospitalized cases | 0.0075 | (0.0074, 0.0076) |  |
|  | $\alpha_{23}$Initial value of transfer rate from confirmed cases to hospitalized cases | 0.0537 | (0.0537, 0.0538) |  |
|  | $\alpha_{33}$Exponential decline rate | 0.0533 | (0.0533, 0.0534) |  |
| $\alpha_{4}(t)$ | Transfer rate from confirmed cases to hospitalized cases in the age group 65-74 years | $\alpha_{4}(t)=\alpha_{14}+\alpha_{24}\exp(-\alpha_{34}t)$ | | MCMC |
|  | $\alpha_{14}$Minimum transfer rate from confirmed cases to hospitalized cases | 0.0208 | (0.0207, 0.0208) |  |
|  | $\alpha_{24}$Initial value of transfer rate from confirmed cases to hospitalized cases | 0.1060 | (0.1060, 0.1061) |  |
|  | $\alpha_{34}$Exponential decline rate | 0.0379 | (0.0378, 0.0380) |  |
| $\alpha_{5}(t)$ | Transfer rate from confirmed cases to hospitalized cases in the age group 75-100 years | $\alpha_{5}(t)=\alpha_{15}+\alpha_{25}\exp(-\alpha_{35}t)$ | | MCMC |
|  | $\alpha_{15}$Minimum transfer rate from confirmed cases to hospitalized cases | 0.0075 | (0.0075, 0.0076) |  |
|  | $\alpha_{25}$Initial value of transfer rate from confirmed cases to hospitalized cases | 0.1096 | (0.1096, 0.1097) |  |
|  | $\alpha_{35}$Exponential decline rate | 0.0034 | (0.0034, 0.0035) |  |
| $\mu_{1}(t)$ | Death rate of hospitalized cases in the age group 0-17 years | $\mu_{1}(t)=\mu_{11}+\mu_{21}\exp(-\mu_{31}t)$ | | MCMC |
|  | $\mu_{11}$Minimum death rate of hospitalized cases | 0.0022 | (0.0022, 0.0023) |  |
|  | $\mu_{21}$Initial value of death rate of hospitalized cases | 0.0037 | (0.0036, 0.0037) |  |
|  | $\mu_{31}$Exponential decline rate | 0.3298 | (0.3298, 0.3299) |  |
| $\mu_{2}(t)$ | Death rate of hospitalized cases in the age group 18-44 years | $\mu_{2}(t)=\mu_{12}+\mu_{22}\exp(-\mu_{32}t)$ | | MCMC |
|  | $\mu_{12}$Minimum death rate of hospitalized cases | 0.0024 | (0.0024, 0.0025) |  |
|  | $\mu_{22}$Initial value of death rate of hospitalized cases | 0.0157 | (0.0157, 0.0158) |  |
|  | $\mu_{32}$Exponential decline rate | 0.0279 | (0.0279, 0.0280) |  |
| $\mu_{3}(t)$ | Death rate of hospitalized cases in the age group 45-64 years | $\mu_{3}(t)=\mu_{13}+\mu_{23}\exp(-\mu_{33}t)$ | | MCMC |
|  | $\mu_{13}$Minimum death rate of hospitalized cases | 0.0047 | (0.0047, 0.0048) |  |
|  | $\mu_{23}$Initial value of death rate of hospitalized cases | 0.0260 | (0.0259, 0.0261) |  |
|  | $\mu_{33}$Exponential decline rate | 9.8077e-04 | (0.0010, 0.0011) |  |
| $\mu_{4}(t)$ | Death rate of hospitalized cases in the age group 65-74 years | $\mu_{4}(t)=\mu_{14}+\mu_{24}\exp(-\mu_{34}t)$ | | MCMC |
|  | $\mu_{14}$Minimum death rate of hospitalized cases | 0.0197 | (0.0196, 0.0197) |  |
|  | $\mu_{24}$Initial value of death rate of hospitalized cases | 0.0456 | (0.0456, 0.0457) |  |
|  | $\mu_{34}$Exponential decline rate | 0.0022 | (0.0021, 0.0022) |  |
| $\mu_{5}(t)$ | Death rate of hospitalized cases in the age group 75-100 years | $\mu_{5}(t)=\mu_{15}+\mu_{25}\exp(-\mu_{35}t)$ | | MCMC |
|  | $\mu_{15}$Minimum death rate of hospitalized cases | 0.0653 | (0.0653, 0.0654) |  |
|  | $\mu_{25}$Initial value of death rate of hospitalized cases | 0.0860 | (0.0860, 0.0861) |  |
|  | $\mu_{35}$Exponential decline rate | 0.0018 | (0.0018, 0.0019) |  |
| $\gamma_{1}$ | Recovery rate of asymptomatic infections in the free environment | 0.4956 | (0.4951, 0.4961) | MCMC |
| $\gamma_{2}$ | Recovery rate of confirmed cases | 0.0673 | (0.0670, 0.0677) | MCMC |
| $\gamma_{3}$ | Recovery rate of hospitalized cases | 0.1045 | (0.1044, 0.1045) | MCMC |
| $\theta_{1}$ | Relative transmission strength of exposed individuals to the symptomatic individuals in the free environment | 0.3633 | (0.3633, 0.3634) | MCMC |
| $\theta_{2}$ | Relative transmission strength of asymptomatic individuals to the symptomatic individuals in the free environment | 0.3143 | (0.3142, 0.3144) | MCMC |
| $\theta_{3}$ | Relative transmission strength of confirmed cases to the symptomatic individuals in the free environment | 0.1006 | (0.1001, 0.1012) | MCMC |
| $\varepsilon$ | The transfer rate from the exposed individuals to the asymptomatic individuals or symptomatic individuals | 0.0533 | (0.0533, 0.0533) | MCMC |
| $k_{1}$ | Increased proportion the contact rate from June 8, 2020 to September 20, 2020 | 2.1707 | (2.1706, 2.1708) | MCMC |
| $k_{2}$ | Increased proportion the contact rate from September 21, 2020 to December 13, 2020 | 4.9980 | (4.9979, 4.9981) | MCMC |
| $E_{1}(0)$ | Initial value of exposed individuals in the age group 0-17 years | 2.1991e+04 | (2.1895e+04, 2.2088e+04) | MCMC |
| $E_{2}(0)$ | Initial value of exposed individuals in the age group 18-44 years | 3.3795e+05 | (3.3788e+05, 3.3802e+05) | MCMC |
| $E_{3}(0)$ | Initial value of exposed individuals in the age group 45-64 years | 4.3447e+05 | (4.3443e+05, 4.3452e+05) | MCMC |
| $E_{4}(0)$ | Initial value of exposed individuals in the age group 65-74 years | 1.0455e+05 | (1.0453e+05, 1.0458e+05) | MCMC |
| $E_{5}(0)$ | Initial value of exposed individuals in the age group 75-100 years | 7.7544e+04 | (7.7464e+04, 7.7623e+04) | MCMC |
| $A_{1}(0)$ | Initial value of asymptomatic individuals in the age group 0-17 years | 1.5480e+03 | (1.4958e+03, 1.6001e+03) | MCMC |
| $A_{2}(0)$ | Initial value of asymptomatic individuals in the age group 18-44 years | 1.7859e+04 | (1.7703e+04, 1.8014e+04) | MCMC |
| $A_{3}(0)$ | Initial value of asymptomatic individuals in the age group 45-64 years | 5.7156e+03 | (5.6644e+03, 5.7669e+03) | MCMC |
| $A_{4}(0)$ | Initial value of asymptomatic individuals in the age group 65-74 years | 6.6935e+03 | (6.6435e+03, 6.7436e+03) | MCMC |
| $A_{5}(0)$ | Initial value of asymptomatic individuals in the age group 75-100 years | 1.8028e+03 | (1.7853e+03, 1.8202e+03) | MCMC |
| $I_{1}(0)$ | Initial value of symptomatic individuals in the age group 0-17 years | 1.9001e+03 | (1.8411e+03, 1.9591e+03) | MCMC |
| $I_{2}(0)$ | Initial value of symptomatic individuals in the age group 18-44 years | 3.9646e+04 | (3.9607e+04, 3.9686e+04) | MCMC |
| $I_{3}(0)$ | Initial value of symptomatic individuals in the age group 45-64 years | 2.7244e+04 | (2.7170e+04, 2.7319e+04) | MCMC |
| $I_{4}(0)$ | Initial value of symptomatic individuals in the age group 65-74 years | 7.5256e+03 | (7.4989e+03, 7.5522e+03) | MCMC |
| $I_{5}(0)$ | Initial value of symptomatic individuals in the age group 75-100 years | 2.6286e+03 | (2.5738e+03, 2.6835e+03) | MCMC |

**Supplementary Table 3. Parameter description and estimated values for models (3) and (4) with vaccination.**

| Parameters | Meanings | Values | 95% CI | Estimation method |
| --- | --- | --- | --- | --- |
| $r$ | The vaccination rate in NYC | 0.0214 | (0.0185, 0.0243) | MCMC |
| $k_{11}$ | Contact rate of the age group 0-17 years made  by age group 0-17 years from December 17, 2020 to January 8, 2021 | 15.5658 | (15.2322, 15.8994) | MCMC |
| $k_{12}$ | Contact rate of the age group 18-44 years made  by age group 0-17 years from December 17, 2020 to January 8, 2021 | 27.8399 | (27.4696, 28.2101) | MCMC |
| $k_{13}$ | Contact rate of the age group 45-64 years made  by age group 0-17 years from December 17, 2020 to January 8, 2021 | 9.0016 | (8.9957, 9.0075) | MCMC |
| $k_{14}$ | Contact rate of the age group 65-74 years made  by age group 0-17 years from December 17, 2020 to January 8, 2021 | 2.3490 | (2.3478, 2.3502) | MCMC |
| $k_{15}$ | Contact rate of the age group 75-100 years made by age group 0-17 years from December 17, 2020 to January 8, 2021 | 8.4779 | (8.4746, 8.4812) | MCMC |
| $k_{22}$ | Contact rate of the age group 18-44 years made by age group 18-44 years from December 17, 2020 to January 8, 2021 | 33.3986 | (32.9748, 33.8223) | MCMC |
| $k_{23}$ | Contact rate of the age group 45-64 years made by age group 18-44 years from December 17, 2020 to January 8, 2021 | 16.6142 | (16.6035, 16.6248) | MCMC |
| $k_{24}$ | Contact rate of the age group 65-74 years made by age group 18-44 years from December 17, 2020 to January 8, 2021 | 3.3879 | (3.3868, 3.3890) | MCMC |
| $k_{33}$ | Contact rate of the age group 45-64 years made by age group 45-64 years from December 17, 2020 to January 8, 2021 | 3.5470 | (3.5456, 3.5483) | MCMC |
| $k_{34}$ | Contact rate of the age group 65-74 years made by age group 45-64 years from December 17, 2020 to January 8, 2021 | 4.3688 | (4.3677, 4.3699) | MCMC |
| $k_{44}$ | Contact rate of the age group 65-74 years made by age group 65-74 years from December 17, 2020 to January 8, 2021 | 1.1549 | (1.1547, 1.1552) | MCMC |
| $\eta_{1}$ | The proportion of symptomatic infections in the age group 0-17 years | 0.0521 | (0.0519, 0.0523) | MCMC |
| $\eta_{2}$ | The proportion of symptomatic infections in the age group 18-44 years | 0.1058 | (0.1055, 0.1061) | MCMC |
| $\eta_{3}$ | The proportion of symptomatic infections in the age group 45-64 years | 0.1531 | (0.1527, 0.1536) | MCMC |
| $\eta_{4}$ | The proportion of symptomatic infections in the age group 65-74 years | 0.2142 | (0.2136, 0.2147) | MCMC |
| $\eta_{5}$ | The proportion of symptomatic infections in the age group 75-100 years | 0.1013 | (0.1011, 0.1014) | MCMC |
| $\delta_{1}$ | Transfer rate from symptomatic individuals to confirmed cases in the age group 0-17 years | 0.0873 | (0.0871, 0.0875) | MCMC |
| $\delta_{2}$ | Transfer rate from symptomatic individuals to confirmed cases in the age group 18-44 years | 0.0614 | (0.0613, 0.0615) | MCMC |
| $\delta_{3}$ | Transfer rate from symptomatic individuals to confirmed cases in the age group 45-64 years | 0.0720 | (0.0716, 0.0723) | MCMC |
| $\delta_{4}$ | Transfer rate from symptomatic individuals to confirmed cases in the age group 65-74 years | 0.0869 | (0.0856, 0.0882) | MCMC |
| $\delta_{5}$ | Transfer rate from symptomatic individuals to confirmed cases in the age group 75-100 years | 0.8728 | (0.8691, 0.8764) | MCMC |
| $\alpha_{41}$ | Minimum transfer rate from confirmed cases to hospitalized cases in the age group 0-17 years | 8.2290e-04 | (0.8008e-03, 0.8450e-03) | MCMC |
| $\alpha_{61}$ | Exponential decline rate in the age group 0-17 years | 0.7717 | (0.7682, 0.7753) | MCMC |
| $\alpha_{42}$ | Minimum transfer rate from confirmed cases to hospitalized cases in the age group 18-44 years | 0.0022 | (0.0021, 0.0022) | MCMC |
| $\alpha_{62}$ | Exponential decline rate in the age group 18-44 years | 0.9837 | (0.9814, 0.9860) | MCMC |
| $\alpha_{43}$ | Minimum transfer rate from confirmed cases to hospitalized cases in the age group 45-64 years | 0.0074 | (0.0073, 0.0074) | MCMC |
| $\alpha_{63}$ | Exponential decline rate in the age group 45-64 years | 0.2176 | (0.2077, 0.2274) | MCMC |
| $\alpha_{44}$ | Minimum transfer rate from confirmed cases to hospitalized cases in the age group 65-74 years | 0.0223 | (0.0222, 0.0224) | MCMC |
| $\alpha_{64}$ | Exponential decline rate in the age group 65-74 years | 0.2635 | (0.2587, 0.2684) | MCMC |
| $\alpha_{45}$ | $\alpha_{15}$Minimum transfer rate from confirmed cases to hospitalized cases in the age group 75-100 years | 0.0092 | (0.0089, 0.0094) | MCMC |
| $\alpha_{65}$ | Exponential decline rate in the age group 75-100 years | 0.0014 | (0.0010, 0.0017) | MCMC |
| $\mu_{41}$ | Minimum death rate of hospitalized cases in the age group 0-17 years | 0.0015 | (0.0014, 0.0015) | MCMC |
| $\mu_{61}$ | Exponential decline rate in the age group 0-17 years | 0.6031 | (0.5857, 0.6204) | MCMC |
| $\mu_{42}$ | Minimum death rate of hospitalized cases in the age group 18-44 years | 0.0025 | (0.0024, 0.0026) | MCMC |
| $\mu_{62}$ | Exponential decline rate in the age group 18-44 years | 0.8653 | (0.8609, 0.8698) | MCMC |
| $\mu_{43}$ | Minimum death rate of hospitalized cases in the age group 45-64 years | 0.0115 | (0.0109, 0.0120) | MCMC |
| $\mu_{63}$ | Exponential decline rate in the age group 45-64 years | 0.3227 | (0.3211, 0.3243) | MCMC |
| $\mu_{44}$ | Minimum death rate of hospitalized cases in the age group 65-74 years | 0.0242 | (0.0233, 0.0251) | MCMC |
| $\mu_{64}$ | Exponential decline rate in the age group 65-74 years | 0.3809 | (0.3792, 0.3826) | MCMC |
| $\mu_{45}$ | Minimum death rate of hospitalized cases in the age group 75-100 years | 0.0492 | (0.0489, 0.0496) | MCMC |
| $\mu_{65}$ | Exponential decline rate in the age group 75-100 years | 0.8864 | (0.8848, 0.8881) | MCMC |
| $m_{11}$ | Contact rate of the age group 0-17 years made  by age group 0-17 years from January 9, 2021 to February 28, 2021 | 35.6195 | (35.2818, 35.9572) | MCMC |
| $m_{12}$ | Contact rate of the age group 18-44 years made  by age group 0-17 years from January 9, 2021 to February 28, 2021 | 32.9952 | (32.6828, 33.3075) | MCMC |
| $m_{13}$ | Contact rate of the age group 45-64 years made  by age group 0-17 years from January 9, 2021 to February 28, 2021 | 7.8019 | (7.7599, 7.8438) | MCMC |
| $m_{14}$ | Contact rate of the age group 65-74 years made  by age group 0-17 years from January 9, 2021 to February 28, 2021 | 1.0164 | (1.0131, 1.0198) | MCMC |
| $m_{15}$ | Contact rate of the age group 75-100 years made by age group 0-17 years from January 9, 2021 to February 28, 2021 | 5.8945 | (5.8685, 5.9205) | MCMC |
| $m_{22}$ | Contact rate of the age group 18-44 years made by age group 18-44 years from January 9, 2021 to February 28, 2021 | 25.2612 | (24.8584, 25.6641) | MCMC |
| $m_{23}$ | Contact rate of the age group 45-64 years made by age group 18-44 years from January 9, 2021 to February 28, 2021 | 4.3878 | (4.3721, 4.4035) | MCMC |
| $m_{24}$ | Contact rate of the age group 65-74 years made by age group 18-44 years from January 9, 2021 to February 28, 2021 | 1.4362 | (1.4203, 1.4521) | MCMC |
| $m_{33}$ | Contact rate of the age group 45-64 years made by age group 45-64 years from January 9, 2021 to February 28, 2021 | 1.0037 | (1.0006, 1.0068) | MCMC |
| $m_{34}$ | Contact rate of the age group 65-74 years made by age group 45-64 years from January 9, 2021 to February 28, 2021 | 0.9737 | (0.9615, 0.9858) | MCMC |
| $m_{44}$ | Contact rate of the age group 65-74 years made by age group 65-74 years from January 9, 2021 to February 28, 2021 | 1.3453 | (1.3301, 1.3605) | MCMC |

**Supplementary Figure 1. Model calibration with the reported cumulative number of confirmed cases.** (a) In the all age groups of NYC. (b) In 0-17 age group. (c) In 18-44 age group. (d) In 45-64 age group. (e) In 65-74 age group. (f) In 75-100 age group.

**Supplementary Figure 2. Model calibration with the reported cumulative number of deaths.** (a) In the all age groups of NYC. (b) In 0-17 age group. (c) In 18-44 age group. (d) In 45-64 age group. (e) In 65-74 age group. (f) In 75-100 age group.

**Supplementary Figure 3. Model calibration with the reported cumulative number of hospitalizations.** (a) In the all age groups of NYC. (b) In 0-17 age group. (c) In 18-44 age group. (d) In 45-64 age group. (e) In 65-74 age group. (f) In 75-100 age group.
